# Differential Gene Expression Profile in the Rat Caudal Vestibular Nucleus is Associated with Individual Differences in Motion Sickness Susceptibility

**DOI:** 10.1371/journal.pone.0124203

**Published:** 2015-04-24

**Authors:** Jun-Qin Wang, Rui-Rui Qi, Wei Zhou, Yi-Fan Tang, Lei-Lei Pan, Yi-Ling Cai

**Affiliations:** Department of Nautical Injury Prevention, Faculty of Navy Medicine, Second Military Medical University, Shanghai, China; Harbin Medical University, CHINA

## Abstract

**Objective:**

To identify differentially expressed genes associated with motion sickness (MS) susceptibility in the rat caudal vestibular nucleus.

**Methods:**

We identified MS susceptible (MSS) and insusceptible (inMSS) rats by quantifying rotation-induced MS symptoms: defecation and spontaneous locomotion activity. Microarray analysis was used to screen differentially expressed genes in the caudal vestibular nucleus (CVN) after rotation. Plasma stress hormones were identified by radioimmunoassay. Candidate genes were selected by bioinformatics analysis and the microarray results were verified by real-time quantitative-PCR (RT-qPCR) methods. By using Elvax implantation, receptor antagonists or recombinant adenovirus targeting the candidate genes were applied to the CVN to evaluate their contribution to MS susceptibility variability. Validity of gene expression manipulation was verified by RT-qPCR and western blot analysis.

**Results:**

A total of 304 transcripts were differentially expressed in the MSS group compared with the inMSS group. RT-qPCR analysis verified the expression pattern of candidate genes, including nicotinic cholinergic receptor (nAchR) α3 subunit, 5-hydroxytryptamine receptor 4 (5-HT_4_R), tachykinin neurokinin-1 (NK_1_R), γ-aminobutyric acid A receptor (GABA_A_R) α6 subunit, olfactory receptor 81 (Olr81) and homology 2 domain-containing transforming protein 1 (Shc1). In MSS animals, the nAchR antagonist mecamylamine significantly alleviated rotation-induced MS symptoms and the plasma β-endorphin response. The NK_1_R antagonist CP99994 and Olr81 knock-down were effective for the defecation response, while the 5-HT_4_R antagonist RS39604 and Shc1 over-expression showed no therapeutic effect. In inMSS animals, rotation-induced changes in spontaneous locomotion activity and the plasma β-endorphin level occurred in the presence of the GABA_A_R antagonist gabazine.

**Conclusion:**

Our findings suggested that the variability of the CVN gene expression profile after motion stimulation might be a putative molecular basis for individual differences in MS susceptibility and provide information for the development of new therapeutic strategies for MSS individuals.

## Introduction

Motion sickness (MS) is a syndrome of autonomic reactions, such as nausea, vomiting, pallor, sweating, increased salivation and stomach awareness, which are commonly provoked by externally imposed motion [1,2]. Currently, the etiology and precise neurobiological mechanism of MS has not been fully clarified and there are several theories interpreting different aspects of MS. The traditional ‘sensory conflict hypothesis’ and ‘neuronal mismatch theory’ suggested that motion sickness may be caused by conflicting auditory, visual and vestibular sensory inputs leading to a mismatch between the actual and the anticipated internal model of the spatial environment [3,4]. According to the ‘postural instability theory’, the occurrence of motion sickness may be associated with preceding unstable postural control at locomotive surroundings on mobile devices [5–7]. However, these theories do not explain apparent individual differences in MS susceptibility, let alone provide detailed information on the underlying molecular bases and mechanisms [8].

As we know, an intact vestibular system is required for MS and serves as an integral component of motion signals in the central nervous system [1,2,9,10]. Vestibular nuclei receive not only vestibular inputs but also somatosensory, proprioceptive, visceral, and visual inputs and motor-related feedback signals [11]. Simultaneously applied vestibular and visual stimulation can reduce the behavioral gain of the vestibular-ocular reflex in mouse and monkey indicating that sensory conflict might be produced in the vestibular nucleus during MS [12,13]. Previous studies have demonstrated that the caudal vestibular nucleus (CVN), including the caudal medial vestibular nucleus (MVe) and the spinal (inferior) vestibular nucleus (SpVe), contribute to both cardiovascular control during head movements and autonomic manifestations of motion sickness through its strong connection with brain stem autonomic areas, such as the solitary tract nucleus and parabrachial nucleus, in a variety of species [14–18]. A variety of provocative environments, such as altered gravito-inertial force, off-axis rotation, centripetal acceleration and space flight, can induce intensive neuronal activation in the CVN as indicated by elevated Fos protein expression [19–22]. Using principal components analysis, a recent study confirmed that neurons in the CVN constitute principal parts of neural networks that contribute to autonomic manifestations, such as retching, excessive salivation, defecation and urination during galvanic vestibular stimulation in felines [23]. Through poly-synaptic connections with the hypothalamic paraventricular nucleus, CVN neurons may also mediate the stress hormone response after vestibular stimulation [24]. In addition, convergence of gastrointestinal afferent signals on CVN neurons can facilitate motion sickness susceptibility in cats exposed to rotation in vertical planes[25]. Based on the idea that neurons in the CVN participate in triggering motion sickness, it is conceivable that they might also contribute to the variability in MS susceptibility.

Many studies have demonstrated that altered gene expression patterns in the VN complex correlate with the properties of VN neurons’ responses to environmental stimulation and with the consequent behavior responses. Altered gene expression patterns were also observed in VN neurons following motion stimulation in the rodents [26–29], yet these observations were not directly connected with MS susceptibility. In the current study, to understand the underlying molecular basis for individual variability in MS susceptibility, we sought to identify differentially expressed genes associated with motion sickness susceptibility in the CVN of male adult rats. Firstly, we established a MS susceptibility animal model by analyzing initial sensitivity to Ferris wheel-like rotation via quantifying two valid MS-related symptoms: defecation during rotation and spontaneous locomotion [30]. The rats’ plasma stress hormone levels were also examined to identify MS susceptibility-related hormonal responses. Then, we identified differentially expressed genes in the CVN between MS susceptible (MSS) and insusceptible (inMSS) animals using microarray analysis. Candidate genes were identified via bioinformatics analysis methods and microarray results were verified by real-time quantitative-PCR (RT-qPCR). Lastly, we examined the relative contribution of these genes to motion sickness susceptibility through functional antagonism or manipulation of gene expression level by using an in vivo Elvax implantation method which is more efficient and convenient in sustained drug delivery over specific brain regions just underneath tissue surface than classical implantation of cannula [31–33].

## Materials and Methods

### 1. Animals and general procedures

Adult male Sprague–Dawley rats weighing 250–300 g were purchased from Shanghai Laboratory Animal Center. The animals were singly housed under a 12 h light: 12 h dark cycle (temperature: 22 ± 2°C and lighting: 8:00–20:00) with free access to food and water. A total of 540 animals were used in this study and all animals were acclimated to the lab environment for 2 weeks before initiation of the experiment and familiarized with the rotation device for 2 hours per day for 3 days prior to the beginning of rotation or static control treatment. The adaptation and rotation procedures were performed during 6:00–10:00 p.m. with the temperature maintained at 22°C.

#### Ethics statement

All surgical procedures were performed under sodium pentobarbital (40 mg/kg, i.p.) anesthesia. All animal protocols and procedures complied with the Guide for the Care and Use of Laboratory Animals (US National Research Council, 1996) and were approved by the Ethics Committee for Animal Experimentation of the Second Military Medical University (Shanghai, PR China). All animal experiments were reported in compliance with ARRIVE guidelines [34,35]. Efforts were made to minimize the number of animals used and the suffering for every animal in each experiment.

#### Rotation device and procedures

The rotation device and detailed rotation methods were described previously [36]. Briefly, the animals were placed in plexiglass containers with the long axis of the body perpendicular to the horizontal rotation rod. The device started to rotate in a clockwise direction at 16°/s^2^ to reach an angular velocity of 120°/s and then began to decelerate at 48°/s^2^ to reach 0°/s. After a 1 s pause, the container continued to rotate in a counter-clockwise direction in the same manner as above. The clockwise-pause-counterclockwise cycle lasted approximately 21 s. All of the rats in the rotation (Rot) groups received 2 hours of rotation stimulation in complete darkness, while the animals in the static control (Sta) groups were kept in the restrainer near the rotation device when Rot animals were being rotated.

#### Tissue preparation

Animals were anesthetized and bilateral CVN (Bregma −11.6mm and −12.3 mm) were dissected and stored following the procedures as our previous study [30]. The precise dissection sites of the CVN tissues were verified by Nissl-staining. Any sample with the edge of incision surface exceeding the boundary of the MVe and SpVe were discarded. Bilateral CVN tissues in one animal were pooled as one sample, frozen on dry ice and stored at −80°C. In Elvax implatation exeriment, half sample from each animal was used for RT-qPCR analysis and another half was used for western blot test.

### 2. Experimental design and grouping

#### Establishment of MS animal model

Sixty rats were used and randomly divided into the following groups: two rotation (Rot) groups received saline (1 ml, 0.9% w/v) or scopolamine (0.1 mg/100 g body weight, i.p.) 30 min before rotation stimulation; two more Rot groups received intratympanic injection of saline (sham-lesioned) or 50–100 μl sodium arsanilate (15 mg in 50 μl saline, chemical labyrinthectomy) 2 weeks before rotation stimulation; and one Sta group (n = 12 in each group). Immediately after rotation or static control treatment, defecation response and spontaneous locomotion activity were evaluated for their validity to be used as indices for assessment of MS symptoms in the rats.

#### MS susceptibility evaluation for microarray experiment

Sixty eight rats were randomly divided into Rot or Sta groups (n = 34 in each group). MSS and inMSS animals were selected from the Rot group after 1st rotation stimulation by quantifying defecation and spontaneous locomotion responses (n = 5 in MSS-Rot and n = 6 in inMSS-Rot). Two weeks later, these animals were re-exposed to rotation and plasma stress hormone levels were tested. Bilateral CVN collections satisfying tissue sampling criteria were further analyzed for differentially expressed genes in microarray experiment.

#### Verification experiment for microarray results

Additional 82 animals was used for screening MSS (n = 12) and inMSS (n = 12) subjects which were then randomly divided into a Rot or Sta group: MSS-Rot, MSS-Sta, inMSS-Rot, or inMSS-Sta (n = 6 in each group). Transcription levels of candidate genes identified in microarray experiment were examined by RT-qPCR test.

#### Pharmacological intervention experiment

Three hundred and thirty animals were used for screening MSS (n = 55) and inMSS subjects (n = 15) which were then divided into drug or adenovirus treatment groups or a sham operation control group (totally 11 MSS and 3 inMSS groups, n = 5 in each group). One week later, Elvax sheets loaded with either receptor antagonists or recombinant adenovirus were implanted over the CVN in MSS or inMSS animals to investigate their effects on motion sickness susceptibility. Animals in the sham operation control group were implanted with control Elvax loaded with solvent. After one week of surgery recovery, MS susceptibility in these animals was re-evaluated and their plasma stress hormone (β-endorphin) concentrations were tested. In adenovirus treated animals, the expression level of candidate genes (Olr81 and Shc1) was also examined by RT-qPCR and western blot test.

### 3. MS susceptibility evaluation

#### MS symptom observation

Immediately after rotation or static control treatment, the animals were taken out of the plexiglass containers of the rotation device and were tested for spontaneous locomotion activity. The number of fecal granules deposited by each animal in the plexiglass container was counted. In the spontaneous locomotion test, locomotion was measured by an animal behavior test system (RD1112-IFO-R-4, Mobiledatum, Shanghai, China). The apparatus consisted of a dark 40 × 40 × 45 cm rectangular chamber with the floor marked with a 16 × 16 grid. The testing was conducted in a soundproof room. The animal was placed in the center of the chamber and left undisturbed for 5 min. Behavior and locomotion tracking of the animals were recorded by an infrared digital video camera. The total distance traveled (dm), immobile (inactivity) duration (s), and body center-point moving (rearing) duration(s) during the 5 min observation were measured with commercially available software (EthoVision XT 8.5, Noldus, Netherlands) [26].

#### MS susceptibility evaluation criteria

**Fig 1:**
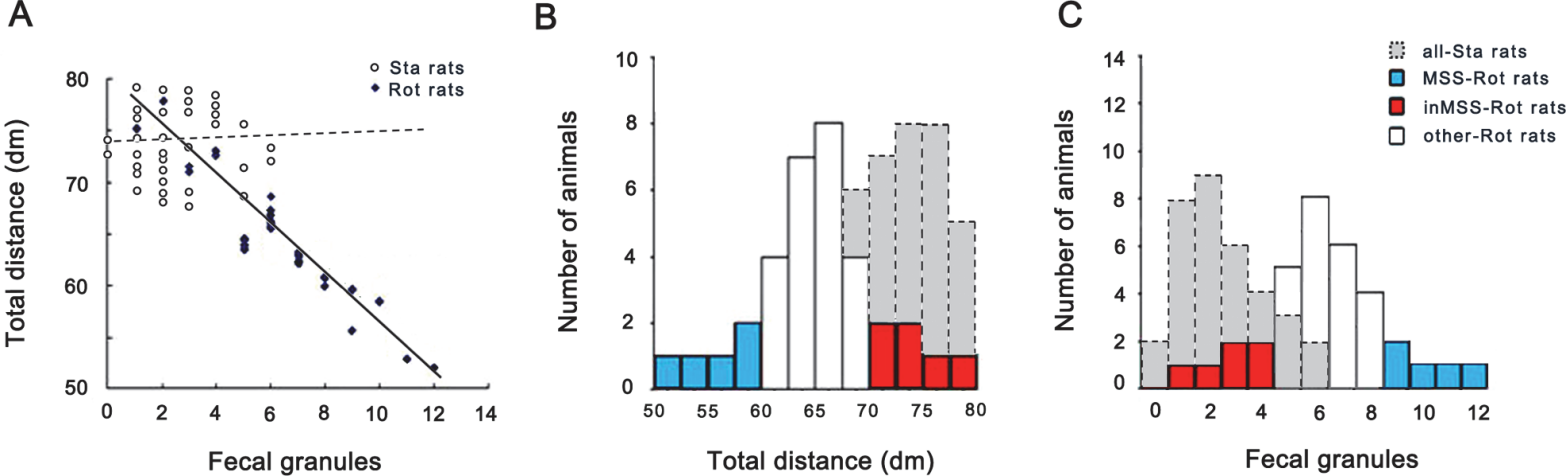
Data analysis for the defecation response and total distance traveled for MS susceptibility evaluation in microarray experiment. (A) Linear relationship between the number of fecal granules and total distance traveled in the Rot and Sta group (n = 34 in each group). The distribution of the number of fecal granules (B) and total distance traveled (C) in staic control (n = 34), MSS-Rot (n = 5), inMSS-Rot (n = 6), and other rotated animals (n = 23).

MS susceptibility evaluation for microarray experiment revealed that there was a strong linear relationship between defecation level and total distance traveled in the Rot group receiving rotation treatment (r = -0.935, F(1,33) = 171.081, p = 0.0001), but not in the Sta group (r = 0.059, F(1,33) = 0.111, p = 0.741) (Fig 1A). Shapiro-Wilk W test analysis showed a normal distribution pattern for the values of defecation level and total distance traveled in the Sta group (W = 0.928, P = 0.028; W = 0.956, P = 0.197) and in the Rot group (W = 0.951, P = 0.106; W = 0.966, P = 0.351) (Fig 1B and 1C). MS susceptibility evaluation criteria was then set as follows: animals in the Rot group with the value of fecal granules distributed within the left 20% of the confidence interval and the value of total distance traveled within the right 20% interval simultaneously were chosen as MSS subjects (n = 5, Fig 1B and 1C blue volume); those with the value for defecation distributed within the left 20% of the confidence interval and the value of total distance traveled within the right 20% of the confidence interval were chosen as inMSS subjects (n = 6, Fig 1B and 1C red volume).

### 4. Molecular biological experiments

#### Microarray analysis

For Affymetrix microarray profiling, total RNA was extracted using an Rneasy Mini Kit following the manufactory’s instructions (Qiagen, German). The GeneChip WT cDNA Synthesis Kit, WT cDNA Amplification Kit, and the WT Terminal Labeling Kit (Affymetrix, Inc., Santa Clara, CA) were used for the cDNA preparation which was hybridized to Rat Exon1.0 ST GeneChip arrays (Affymetrix, America) according to the Users’ Manuals. Affymetrix Expression Console Software (version 1.1.2) was used for microarray analysis. RVM t-test was applied to filter the differentially expressed genes between MSS-Rot and inMSS-Rot group. Fold-change was calculated as the ratio between the average values of gene expression in MSS-Rot relative to inMSS-Rot animals. Two-dimensional hierarchical clustering of the expression data was performed using a Pearson correlation distance matrix and average linkage clustering. Gene ontology (GO) analysis was applied to analyze the main functions of differentially–expressed genes. Pathway analysis was used to identify the significant pathways according to KEGG, Biocarta and Reatome databases via Fisher’s exact test and the *χ* ^2^ test. The threshold of significance was defined by P-value at 0.05 and the screening condition was set as false discovery rate (FDR) under 5%. All microarray datasets were submitted to the ArrayExpress repository (http://www.ebi.ac.uk/arrayexpress/experiments/E-MTAB-3213).

#### RT-qPCR test

Total RNA extraction procedure was the same as in the microarray experiment. The RT-qPCR reactions were conducted in a Rotor-Gene (RG-3000A, Corbett Research) PCR machine. The amount of cDNA per sample was determined using a SYBR Premix Ex Taq kit (Takara). Progression of the PCR reaction was assessed by changes of the SYBR Green dye fluorescence attached to double-stranded DNA. All values were normalized to the housekeeping gene glyceraldehyde phosphate dehydrogenase (GAPDH). The primers used for real-time PCR are shown in S1 Table.

#### Western blot test

Wetern blot analysis was performed as previously described [30]. The primary antibodies used in this study were anti-Olr81 (1:1000; Santa Cruz Biotechnology, Santa Cruz, CA, USA) and anti-Shc1 (1:1000; Cell signaling, Beverly, MA, USA). The secondary antibodies used were peroxidase-labeled anti-goat IgG and anti-rabbit IgG (all from Jackson, West Groove, PA, USA) at 1:5000 dilution. Signal intensities of Olr81 and Shc1 bloting bands were normalized against the internal control (GAPDH).

#### Plasma hormone measurements

Blood was collected immediately after decapitation and the plasma was separated and stored at −80°C for further analyses. Plasma epinephrine, norepinephrine, arginine-vasopressin (AVP), adrenocorticotropic hormone (ACTH) and β-endorphin levels were measured by radioimmunoassay following the instructions in the kits generously provided by Prof. Zhao XL at the Second Military Medical University or purchased from North Institute of Biological Technology Co (Beijing, China).

### 5. In vivo Elvax implantation technology

#### Recombinant adenovirus preparation

Recombinant adenovirus for the over-expression of Shc1 (pAd-Shc1) was generated as follows. The rat Shc1 gene was synthesized de novo by rapid polymerase chain assembly and cloned into the SpeI-SgsI site of pENTR-IRES-EGFP (Invitrogen). The adenoviral plasmid pAd-CMV-Shc1-IRES-EGFP was generated by LR clonase-mediated recombination using pAd-CMV-V5-DEST (Invitrogen) as the acceptor and the pENTR-Shc1-IRES-GFP (Invitrogen) as the donor. Recombinant adenoviruses were propagated in HEK293 cells and purified using the Adenovirus Purification Miniprep Kit (Biomiga V1160) following the manufacturer’s instructions.

The Olr81 targeting recombinant adenoviral pAd-miOlr81 was generated by cloning synthetic oligonucleotides encoding complimentary miRNAs for rat Olr81 mRNA into the pENTR-miR vector (Invitrogen), followed by homologous recombination with pAd-CMV-V5-DEST (Invitrogen). The sequence for the oligonucleotides was: 5’- TGCTGAGGAATGTGCTATTACATGAGGTTTTGGCCACTGACTGACCTCATGTAAGCACATTCCT -3’.

#### Elvax preparation and implantation

**Fig 2:**
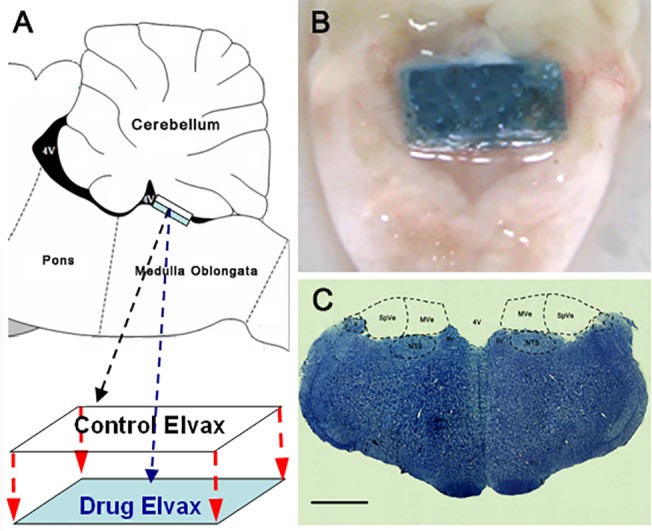
Elvax preparation and CVN tissue sampling. (A) Illustration for Elvax sheet preparation and (B) photograph of a rat brain stem showing the location of Elvax sheet implantation. (C) Representative Nissl-staining image showing the CVN sampling region. 4V: fourth ventricle; MVe: medial vestibular nucleus; SpVe: spinal vestibular nucleus; NTS: nucleus of solitary tract; Pr: prepositus nucleus; X: nucleus X; Bar: 200 μm.

ELVAX has been successfully used to deliver water or dimethylsulfoxide (DMSO) soluble drug onto rodent’s brain surface [31,32]. In this experiment, Elvax sheets were prepared following the procedures described by Nodal FR [32]. Briefly, plastic beads of the ethylene–vinyl acetate copolymer Elvax 40-W (Elvax 40P; Du Pont) were washed in several changes of 95% and 100% alcohol for 24 h. After the beads were dried, they were dissolved in methylene chloride (0.15 g/1.5 ml solvent). Drugs dissolved in either 50 μl of distilled water (mecamylamine, CP99994 or gabazine) or 50 μl of DMSO (0.03% in final concentration, RS39604) were added to the Elvax mixture. The final embedded concentrations of the agents were as follows: mecamylamine, 1.25 mM or 2.50 mM; CP99994 and RS39604, 5 mM or 10 mM; and gabazine, 0.25 mM or 0.50 mM. Mecamylamine and gabazine were purchased from Sigma-Aldrich (St. Louis, MO, USA). CP99994 and RS39604 were purchased from Tocris (Tocris, UK). For in vivo adenovirus infection, 100 or 200 μl of either pAd-miOlr81 or pAd-Shc1 was added to the Elvax mixture. The final embedded titers were 3.50 ×10^7^ GFU/ml or 7.00 ×10^7^ GFU/ml for pAd-miOlr81 and 3.35 ×10^8^ GFU/ml or 6.70 ×10^8^ GFU/ml for pAd-Shc1. For the sham control experiment, control Elvax sheets containing only the vehicle (water, DMSO or culture medium) were also prepared. To visualize the implantation, 25 μl of 5% Fast Green was also added to the Elvax mixture. The mixture was then vortexed, frozen quickly in a dry ice/acetone bath, and transferred to a pre-chilled glass petri dish and freeze-dried overnight (−60°C, 10^–4^ atm). All dry Elvax mixtures (approximately 200 μm thick) were cut into 4 mm×1.5 mm×200 μm rectangular sheets. To avoid contaminating the cerebellum, two-layer Elvax sheets were prepared by covering an agent-loaded Elvax sheet (lower layer) with a solvent control Elvax sheet (upper layer) and stored on filter paper at 4°C prior to implantation (Fig 2A).

For the Elvax implantation surgery, animals were anesthetized and placed on a stereotaxic frame (Narishige, Japan). The skin and muscles of the neck were incised and dissected along the midline dorsally to expose the atlanto-occipital membrane. Then, a partial occipital craniotomy was performed and the cerebellum was pushed forward to expose the underlying fourth ventricle. A two-layer Elvax sheet was then placed over the CVN region, covering most of its surface (Fig 2B). After the cerebellum was repositioned completely, the opening in the skull was closed with dental cement and the overlying neck muscles and skin were sutured. Animals received postsurgery antibiotics penicillin (400000U/kg, i.p.) and analgesics ibuprofen (30mg/kg, in the drinking water) for 3 days.

### 6. Statistical analysis

All statistical analyses were conducted with the SPSS v13.0 statistical program and data are expressed as the mean ± S.D. One-way ANOVA analysis was performed to examine the difference among groups in the MS animal model establishment experiment and the differences following pharmacological intervention in the β-endorphin concentration and gene expression (Olr81 and Shc1) test. Fisher’s LSD post hoc test was used to analyze the difference between each group when a significant main effect was obtained. Pearson correlation analysis was conducted to determine if there was a linear relationship between the defecation level and the total distance traveled for MS susceptibility evaluation in microarray experiment. Normal distribution for these indices was constituted and evaluated by Shapiro-Wilk analysis. A t-test analysis was performed to examine the difference in MS symptoms and plasma hormone levels between MSS and inMSS animals in MS susceptibility evaluation for microarray experiment and the difference in β-endorphin levels between the sham MSS and the sham inMSS group in Elvax experiment. Two-factorial analysis of variance (ANOVA) for repeated-measures was performed using the General Linear Protocol to examine the effect of susceptibility and rotation on the mRNA levels for candidate genes in the RT-qPCR verification experiment and the effect of time, drug concentration (virus titer) or susceptibility on MS symptoms in the pharmacological intervention experiment. Boferoni post hoc test was used to analyze the difference between each group when a significant main effect or interaction effect was obtained. The level of significance was set at p<0.05.

## Results

### 1. MS susceptibility evaluation

#### Establishment of MS animal model

Table 1 show that rotation stimulation leads to an increase in defecation and a decrease in spontaneous locomotion activity (hypoactivity) in Rot animals receiving saline (i.p.) and sham lesion treatment compared with Sta controls. Defecation response was significantly decreased in Rot animals receiving scopolamine administered prior to rotation stimulation compared to those receiving saline treatment. Bilateral labyrinthectomy also significantly reduced defecation in Rot animals compared to the sham-lesioned group (P<0.05). Both scopolamine administration and bilateral labyrinthectomy significantly alleviated rotation-induced decreases in total distance traveled and center-point moving duration and the duration of immobility decreased in Rot animals compared to the saline and sham-lesioned group, respectively (P<0.05).

**Table 1:** MS symptoms observed in animals receiving static control or rotation treatment.

	Sta group	Rot groups			
		Saline	Scopolamine (0.1 mg/100 g)	Sham-lesioned	Chemical labyrinthectomy
No. of fecal granules	2.42 ± 1.24	7.08 ± 2.27 Δ	2.91 ± 1.67 *	7. 75 ± 2.17 Δ	3.00 ± 1.41 †
Total distance (dm)	72.75 ± 4.07	65.38 ± 5.69 Δ	74.17 ± 4.43 *	65.63 ± 5.46 Δ	73.50 ± 5.20 †
Immobile duration (s)	141.67 ± 4.75	146.75 ± 7.07 Δ	142.42 ± 5.16 *	147.33 ± 6.77 Δ	140.75 ± 5.75 †
Center-point moving (s)	97.08 ± 3.68	89.75 ± 6.96 Δ	96.58 ± 3.75 *	89.92 ± 7.39 Δ	98.50 ± 4.81 †

Δ P<0.05 compared with Sta group

* P<0.05 compared with Rot-saline group

† P<0.05 compared with Rot-sham operation group.

#### Behavioral and hormonal responses in MSS and inMSS animals

T-test analysis showed that defecation levels and immobility duration were significantly increased and the total distance traveled and center-point moving duration were decreased in the MSS group compared to the inMSS group in MS susceptibility evaluation experiment prior to microarray analysis. There was also a significant increase in plasma β-endorphin levels and an insignificant trend of increased AVP and ACTH levels in MSS animals (Table 2).

**Table 2:** MS symptoms and microarrayed plasma hormone levels of MSS-Rot and inMSS-Rot groups.

	MSS animals (n = 5)	inMSS animals (n = 6)
**MS symptoms**		
No. of fecal granules	10.80 ± 1.09 **	3.33 ± 1.03
Total distance (dm)	55.99 ± 3.61 **	74.73 ± 3.91
Immobile duration (s)	154.52 ± 6.03 **	140.36 ± 4.62
Center-point moving (s)	85.16 ± 8.21 *	98.30 ± 4.96
**Plasma hormones**		
Epinephrine(ng/L)	93.97 ± 12.13	93.89 ± 12.77
Noradrenaline (ng/L)	157.57 ± 26.95	146.90 ± 28.77
AVP(pg/ml)	501.54 ± 131.99	416.03 ± 169.30
β-endorphin (pg/ml)	106.01 ± 54.94 *	51.47 ± 26.54
ACTH(pg/ml)	204.04 ± 86.78	159.03 ± 109.09

**P<0.01

* P<0.05 compared with inMSS-Rot group

### 2. MS susceptibility-associated genes

#### Candidate genes identified by microarray analysis

**Fig 3:**
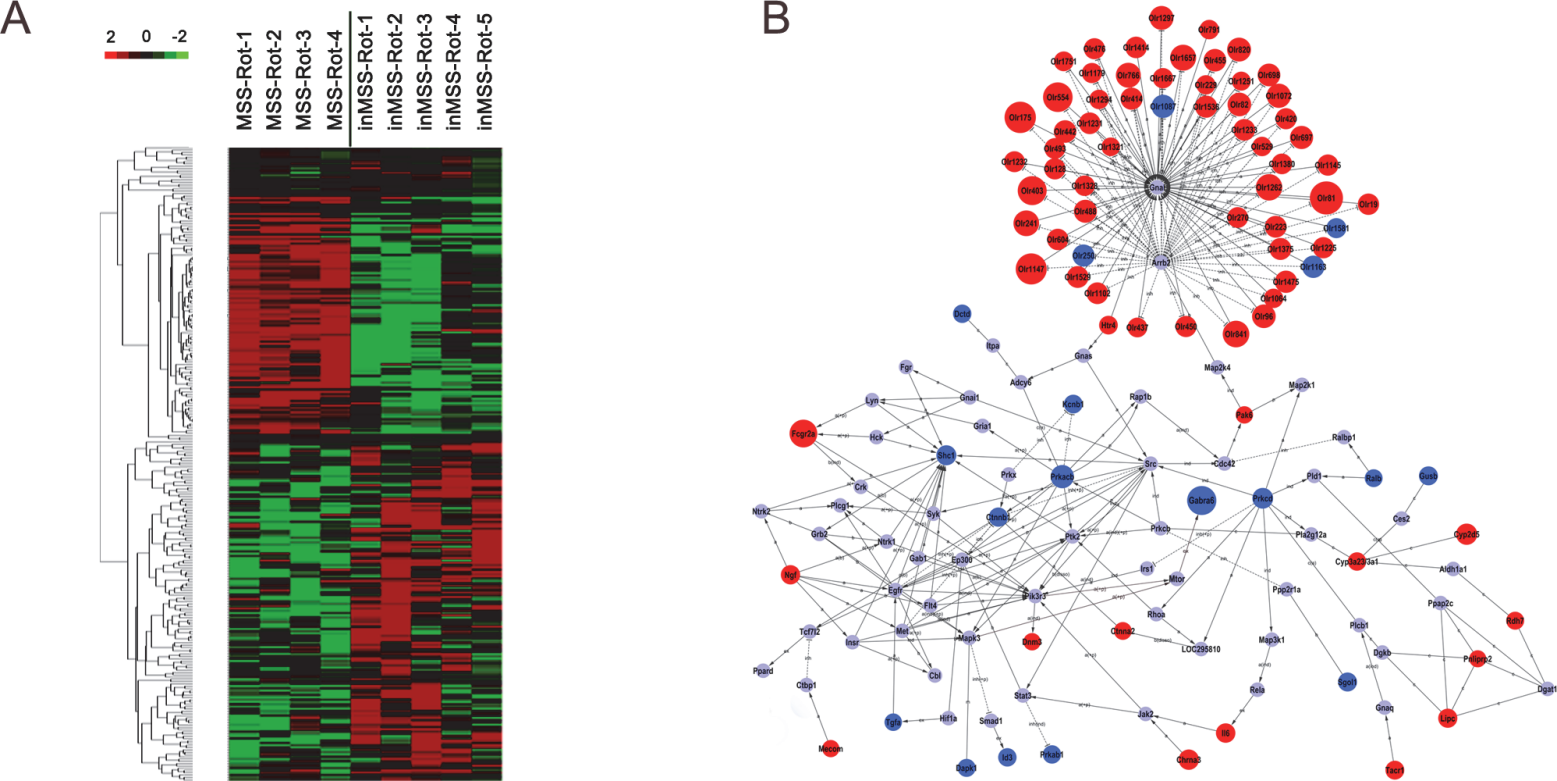
Hierarchical clustering of the signal value and signaling regulatory network of the differentially expressed genes. (A)Heat map of differential changes in gene expression between MSS-Rot and inMSS-Rot animals. The dendrogram was produced by hierarchical clustering of the signal value of differentially expressed genes in MSS-Rot rats (n = 4, left) and inMSS-Rot samples (n = 5, right). Colorimetric scaling of standardized gene expression values ranging from low (green) to high (red) is shown in the legend (upper left). (B) Signal-net of the differentially expressed genes. Red nodes and blue nodes represent up-regulated and down-regulated genes, respectively. Grey nodes represent intermediate genes functionally connecting the differentially expressed genes. Solid lines with arrow head indicate activation (a), dotted lines indicate inhibition (inh) and solid lines indicate binding (b) or expression regulation (ex). Node size represents the degrees of diiference in gene expression levels of MSS relative to inMSS.

CVN tissue samples, which satisfied the sampling criteria (Fig 2C), were included in the following microarray experiment (n = 4 in MSS-Rot and n = 5 in inMSS-Rot). Hierarchal clustering analysis revealed that gene differences can be successfully distinguished between the MSS-Rot and inMSS-Rot groups (Fig 3A). Microarray analyses identified a total of 304 transcripts differentially expressed in the MSS-Rot group compared to the inMSS-Rot group: 207 transcripts were relatively up-regulated and 97 transcripts were down-regulated. The top 45 up-regulated genes (MSS-Rot/inMSS-Rot SS-Rot/inMSS-Roted g7 down-regulated genes (MSS-Rot/inMSS-Rot SS-Rot/ are listed in S2 and S3 Tables. Additionally, signal-net analysis integrated the up-regulated and down-regulated genes and delineated the signaling regulatory networks of their expression products (Fig 3B).

Gene ontology (GO) hierarchy analysis revealed significant functions, including the positive regulation of cholinergic synaptic transmissions (P = 0.0097, enrichment = 205.06), response to electrical stimulus (p = 0.00028, enrichment = 102.53), the G protein coupled receptor (GPCR) protein signaling pathway (P = 0.034, enrichment = 57.95), the response to nutrient levels (P = 0.00070, enrichment = 68.35), the response to calcium ions (P = 0.0049, enrichment = 11.19), the response to organic cyclic substances (P = 0.000054, enrichment = 10.34), the response to peptide hormone stimuli (P = 0.0021, enrichment = 9.01) and GABAergic synaptic transmission (P = 0.029, enrichment = -67.61). Among these GO categories, we found four differentially expressed neurotransmitter receptor genes: Chrna3-nicotinic cholinergic receptor (nAchR) α3 subunit; Htr4 -5-hydroxytryptamine (serotonin) receptor 4 (5-HT_4_R); Tacr1-tachykinin neurokinin-1 receptor (NK_1_R) and Gabra6 (γ-aminobutyric acid A receptor [GABA_A_R] α6 subunit). Gabra6 was also the most statistically significant down-regulated gene in MSS animals.

Among the differentially expressed genes, we found 51 up-regulated genes and 4 down-regulated genes belonging to the olfactory receptor (OR) superfamily. We then analyzed the evolutionary properties of the gene sequences and found 5 “fish-like” (Class I) OR genes (Olr81, Olr82, Olr96, Olr128, Olr175), which are more conserved among species and more evolutionarily ancient than tetrapod (Class II) ORs (the remaining 50 ORs) [37,38]. Furthermore, by checking the Gene database on PubMed, we found that Olr81 is homologous to OR52J3 in humans [www.ncbi.nlm.nih.gov/homologene/66215]. Additionally, pathway analysis revealed 3 up-regulated and 7 down-regulated pathways identified from the KEGG database (p<0.05). The most significantly up-regulated pathway was the olfactory transduction pathway and the most down-regulated pathway was the insulin signaling pathway (S4 Table.). The most statistically significant genes in these two pathways were Olr81 (P = 0.0035789) and homology 2 domain containing transforming protein 1 (Shc1) (P = 0.0366554). We finally selected Olr81, Shc1, Chrna3, Htr4, Tacr1 and Gabra6 as the candidate genes for the following experiment.

#### Verification of candidate genes by RT-qPCR

**Fig 4:**
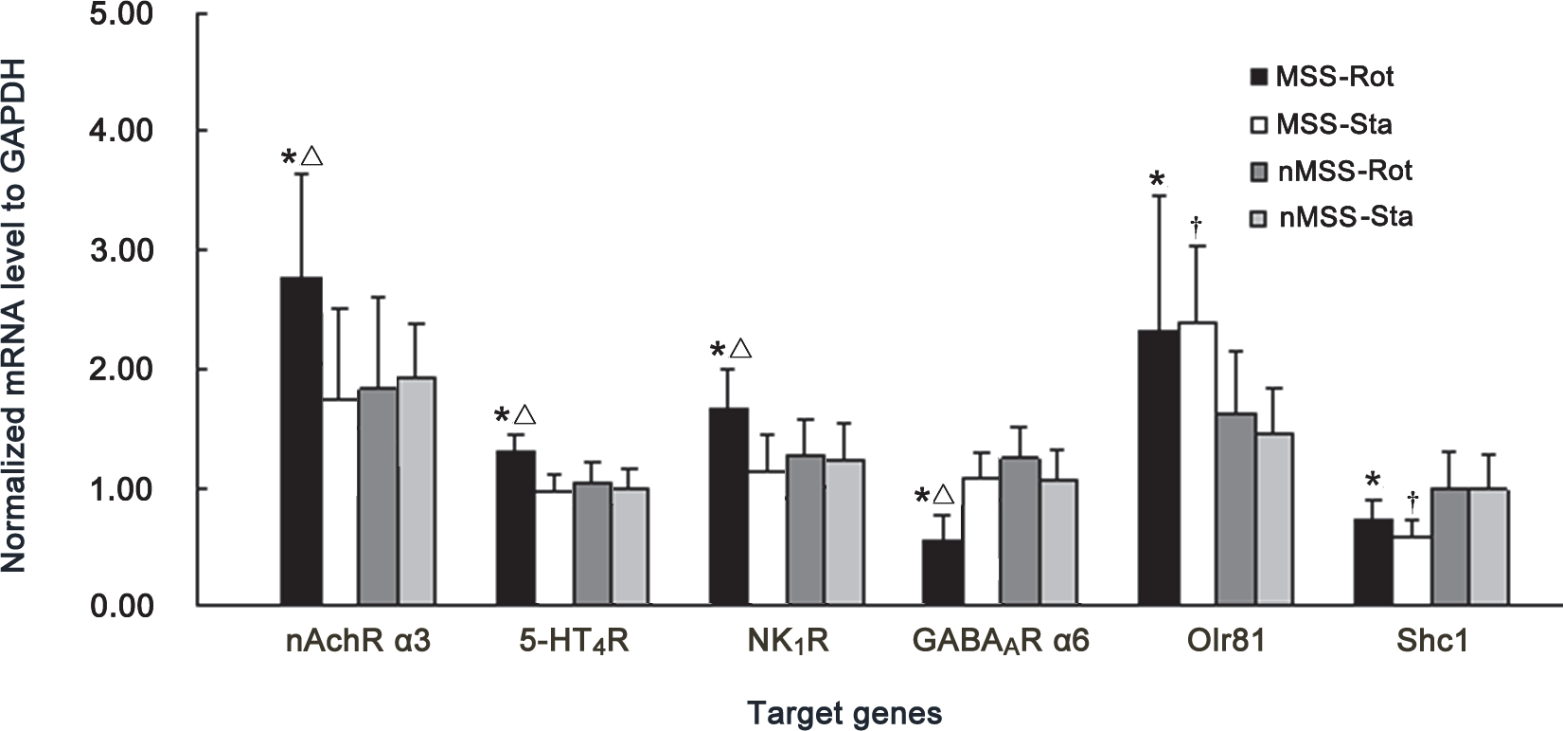
RT-qPCR verification of candidate genes mRNA levels, including the nAchR α3 subunit, 5-HT_4_R, NK_1_R, the GABA_A_R α6 subunit, Olr81 and Shc1 in the CVN of MSS and inMSS rats receiving 2 hours of rotation or static treatment. Values in each group (n = 6) are expressed as the percentage of their corresponding GAPDH values (100%) and shown as the means (±S.E.). *P<0.05, compared with inMSS-Rot group; ΔP<0.05, compared with MSS-Sta group; † P<0.05 compared with inMSS-Sta group.

All tissue samples satisfied the sampling criteria and were further analyzed in this experiment. The MSS-Rot/ inMSS-Rot ratio of gene expression for the nAchR α3 subunit, 5-HT_4_R, NK_1_R, the GABA_A_R α6 subunit, Olr81 and Shc1 were similar to the ratios obtained by microarray analysis (S5 Table.). A 2 (susceptibility difference) × 2 (rotation condition) factorial ANOVA analysis revealed significant effects of susceptibility [F (1, 20) = 4.616, P = 0.047] and rotation [F (1, 20) = 4.744, P = 0.045] and a susceptibility×rotation interaction [F (1, 20) = 6.915, P = 0.018] on nAchR α3 subunit expression; significant susceptibility [F (1, 20) = 8.237, P = 0.011] and rotation [F (1, 20) = 10.184, P = 0.006] effects and a susceptibility×rotation interaction [F (1, 20) = 4.861, P = 0.042] on 5-HT_4_R expression; significant susceptibility [F (1, 20) = 6.318, P = 0.023] and rotation [F (1, 20) = 8.995, P = 0.008] effects and a susceptibility×rotation interaction [F (1, 20) = 5.985, P = 0.026] on NK_1_R expression; significant susceptibility [F (1, 20) = 6.096, P = 0.025] and rotation [F (1, 20) = 22.003, P = 0.0001] effects and a susceptibility×rotation interaction [F (1, 20) = 25.303, P = 0.0001] on GABA_A_R α6 expression. Boferoni post hoc analysis revealed that the mRNA levels of the nAchR α3 subunit, 5-HT_4_R, and NK_1_R were increased, while the GABA_A_R α6 subunit was decreased in MSS-Rot group compared to the MSS-Sta and inMSS-Rot groups (P<0.05). Meanwhile, there was a significant susceptibility effect on Olr81 [F (1, 20) = 5.139, P = 0.035] and Shc1 [F (1, 20) = 9.153, P = 0.007] expression. The Olr81 mRNA level was increased and the Shc1 mRNA level was decreased in the MSS-Rot and MSS-Sta groups when compared with the corresponding inMSS animals (P<0.05, Fig 4).

### 3. Effects of Elvax implantation on MS susceptibility

#### Validity of adenovirus delivery

One-way ANOVA analysis found a significant effect of pAd-miOlr81 delivery on Olr81 mRNA level (P = 0.047), and a significant effect of pAd-Shc1 treatment on Shc1 mRNA level (P = 0.002) in the CVN of MSS animals. LSD post hoc analysis showed that pAd-miOlr81, at the high final titer in Elvax (7.00 ×10^7^ GFU/ml), significantly decreased Olr81 mRNA level (P = 0.015), while pAd-Shc1, at both final titers (3.35 ×10^8^ GFU/ml and 7.00 ×10^8^ GFU/ml in Elvax), significantly enhanced Shc1 transcription (P = 0.011 and 0.001) in MSS animals compared to sham operation controls (S1 Fig). There was also a significant effect of pAd-miOlr81 treatment which decreased Olr81 protein level at 7.00 ×10^7^ GFU/ml (P = 0.001), and a significant effect of pAd-Shc1 treatment which significantly increased Shc1 protein level at both final titers (P = 0.001, S1 Fig).

#### Defecation response

**Fig 5:**
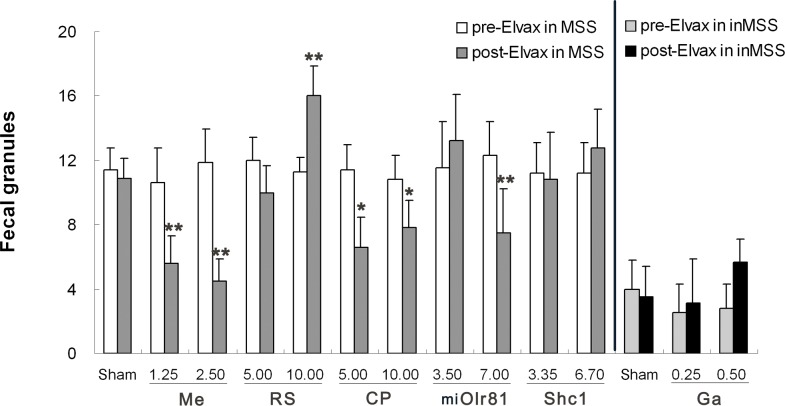
Effects of Elvax implantation over the CVN on the defecation response induced by rotation stimulation. In MSS animals, Elvax was loaded with solvent (Sham); 1.25 or 2.50 mM mecamylamine (Me); 5.00 or 10.00 mM RS39604 (RS); 5.00 or 10.00 mM CP99994 (CP); 3.50×10^7^ or 7.00 ×10^7^ GFU/ml pAd-shOlr81 (shOlr81); or 3.35 ×10^8^ or 6.70 ×10^8^ GFU/ml pAd-Shc1 (Shc1) at final concentration/titer (n = 5 in each group). In inMSS animals, Elvax was loaded with solvent (Sham) or gabazine (Ga) to a final concentration of 0.25 or 0.5 mM (n = 5 in each group). All data presented are expressed as the means and the vertical bars represent SEM. Statistical significance: **P<0.01, * P<0.05 compared with the rotation-induced defecation response before Elvax implantation.

In sham operation control animals, two-way ANOVA analysis showed no effect of time [F (1, 19) = 1.005, P = 0.331], but a significant effect of susceptibility [F (1, 19) = 88.804, P = 0.001] and time×susceptibility interaction [F (1, 19) = 5.789, P = 0.029] on defecation levels. Boferoni post hoc analysis revealed that defecation levels remained unchanged after control-Elvax implantation in the MSS sham group and the inMSS sham group compared to their pre-Elvax implantation levels, but they were much higher in the MSS sham group than in the inMSS sham animals (P = 0.001, Fig 5).

In MSS animals, two-way ANOVA analysis found no effect of time [F (1, 29) = 2.251, P = 0.147], but a significant effect of concentration [F (2, 29) = 7.142, P = 0.004] and a time×concentration interaction [F (2, 29) = 4.861, P = 0.042] on defecation levels after mecamylamine-Elvax implantation. Boferoni post hoc analysis found that mecamylamine treatment, at both concentrations (1.25 mM and 2.50 mM in Elvax), significantly deceased the rotation-induced defecation response compared with the corresponding pre-Elvax level, in a dose-dependent manner (P = 0.0019 and 0.0011). There was a significant effect of concentration [F (2, 29) = 3.871, P = 0.0425] and a time×concentration interaction [F (2, 29) = 4.105, *p =* 0.0363] on defecation levels after RS39604-Elvax implantation. CP99994-Elvax implantation resulted in a significant effect of concentration [F (2, 29) = 8.735, *p =* 0.00187] and a time×concentration interaction [F (2, 29) = 5.648, P = 0.0113]. RS39604 treatment, at a concentration of 10 mM, significantly increased the rotation-induced defecation response compared with pre-treatment levels (P = 0.0037). CP99994 treatment, at both concentrations (5 mM and 10 mM in Elvax), significantly deceased the rotation-induced defecation response (P = 0.0016 and 0.049, Fig 5).

There is also a significant effect of time [F (1, 29) = 0.942, P = 0.342], titer [F (2, 29) = 8.426, P = 0.0019] and a time×titer [F (2, 29) = 9.976, P = 0.00082] interaction on the defecation response after pAd-miOlr81 infection, which at the high final titer in Elvax (7.00 ×10^7^ GFU/ml) alleviated the defecation response to rotation in MSS animals when compared to their pre-Elvax treatment data (P = 0.0062); No significant effects were observed after pAd-Shc1 treatment.

In inMSS animals, no significant effect of time, concentration or time×concentration interaction on the defecation response was observed after gabazine-Elvax implantation, while an insignificant trend towards increased defecation at a higher concentration (5.0 mM in Elvax) was found (P = 0.067).

#### Spontaneous locomotion activity

Two-way ANOVA analysis found a significant effect of susceptibility on the total distance traveled [F (1, 19) = 20.756, P = 0.001], duration of immobile [F (1, 19) = 29.309, P = 0.001] and center-point moving duration [F (1, 19) = 27.995, P = 0.001] in sham operation control animals. Boferoni post hoc analysis revealed that immobility duration was significantly increased and the total distance traveled and center-point moving duration were decreased in the MSS sham group compared to the inMSS sham group (P<0.05), while there was no difference in these indices between pre-Elvax and post-Elvax level in either the MSS sham or the inMSS sham animals.

**Fig 6:**
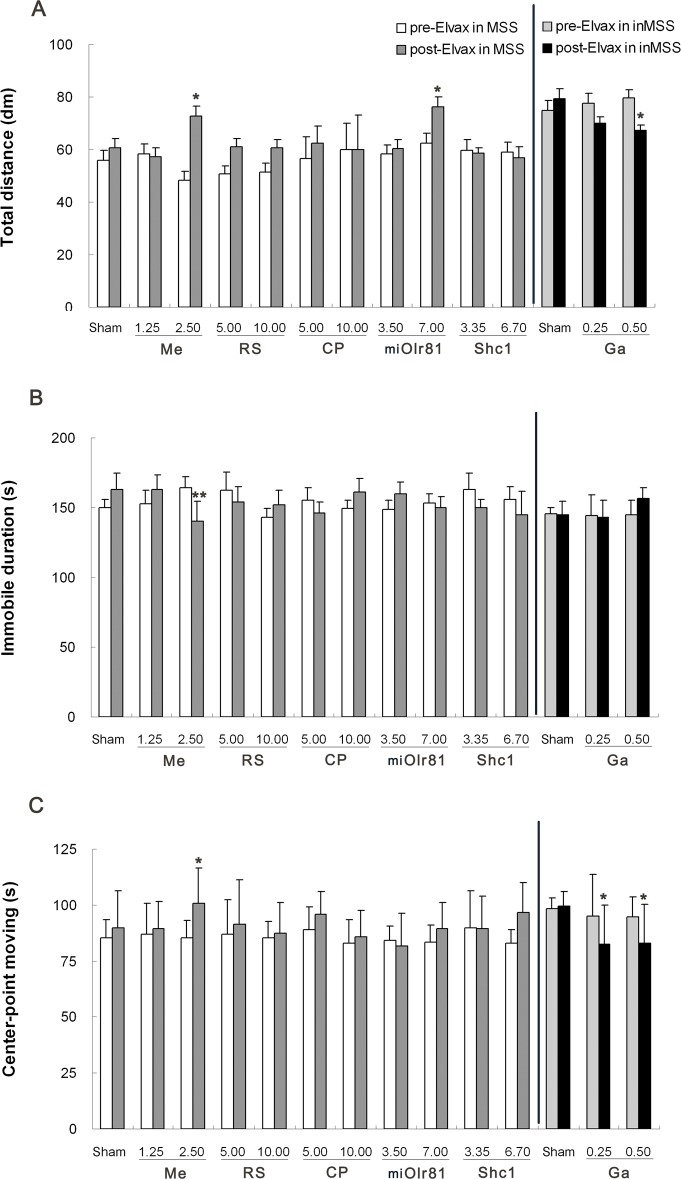
Effects of Elvax implantation over the CVN on spontaneous locomotion activity after rotation stimulation. (A) Total distance travelled; (B) Immobile duration; (C) Body center-point moving duration. The final concentration of mecamylamine (Me), RS39604 (RS, CP99994 (CP) and gabazine (Ga) and the final titer of pAd-shOlr81 (shOlr81) and pAd-Shc1 (Shc1) in Elvax, and the number of animals used in each treatment group are the same as those in Fig 5. All data presented are expressed as the means and the vertical bars represent SEM. * P<0.05 compared with rotation-induced spontaneous locomotion activity response before the Elvax implantation.

There was a significant time×concentration interaction on the total distance traveled [F (2, 29) = 5.067, P = 0.035] and immobility duration [F (2, 29) = 10.715, P = 0.0004] and a significant effect of concentration [F (1, 29) = 4.085, P = 0.029] on center-point moving duration after mecamylamine-Elvax implantation. Mecamylamine treatment, at a concentration of 2.50 mM in Elvax, increased the total distance traveled (P = 0.0233, Fig 6A) and the center-point moving duration (P = 0.0467, Fig 6C) and decreased immobile duration (P = 0.0054, Fig 6B) when compared with pre-Elvax treatment level. No significant effects of time, concentration or time×concentration interaction on the total distance traveled, the duration of immobility and center-point moving duration were observed after RS39604, CP99994 or pAd-Shc1 treatment. There was a significant time×concentration interaction [F (2, 29) = 3.811, P = 0.0379], but no effect of time [F (1, 29) = 0.506, P = 0.484] or titer [F (2, 29) = 1.956, P = 0.165] on the total distance traveled after pAd-miOlr81 Elvax implantation, and no significant effect on the duration of immobility or center-point moving duration was observed. Boferoni post hoc analysis found that pAd-miOlr81 treatment at 7.00 ×10^7^ GFU/ml significantly relieved the rotation-induced decrease in total distance traveled (P = 0.025, Fig 6A).

In inMSS animals, a significant concentration effect [F (2, 29) = 4.308, P = 0.050] on the total distance traveled and a significant effect of time×concentration [F (2, 29) = 3.528, P = 0.0468] interaction on the duration of immobility were observed after gabazine-Elvax implantation. Gabazine treatment, at both concentrations (2.5 mM and 5.0 mM in Elvax), significantly decreased the total distance traveled (Fig 6A), the center-point moving duration (P = 0.047 and 0.037, Fig 6C), and slightly increased the duration of immobility (P = 0.053) at 5.0 mM in Elvax (Fig 6B) in inMSS animals exposed to rotation compared with their pre-Elvax treatment levels.

#### Plasma β-endorphin concentration

**Fig 7:**
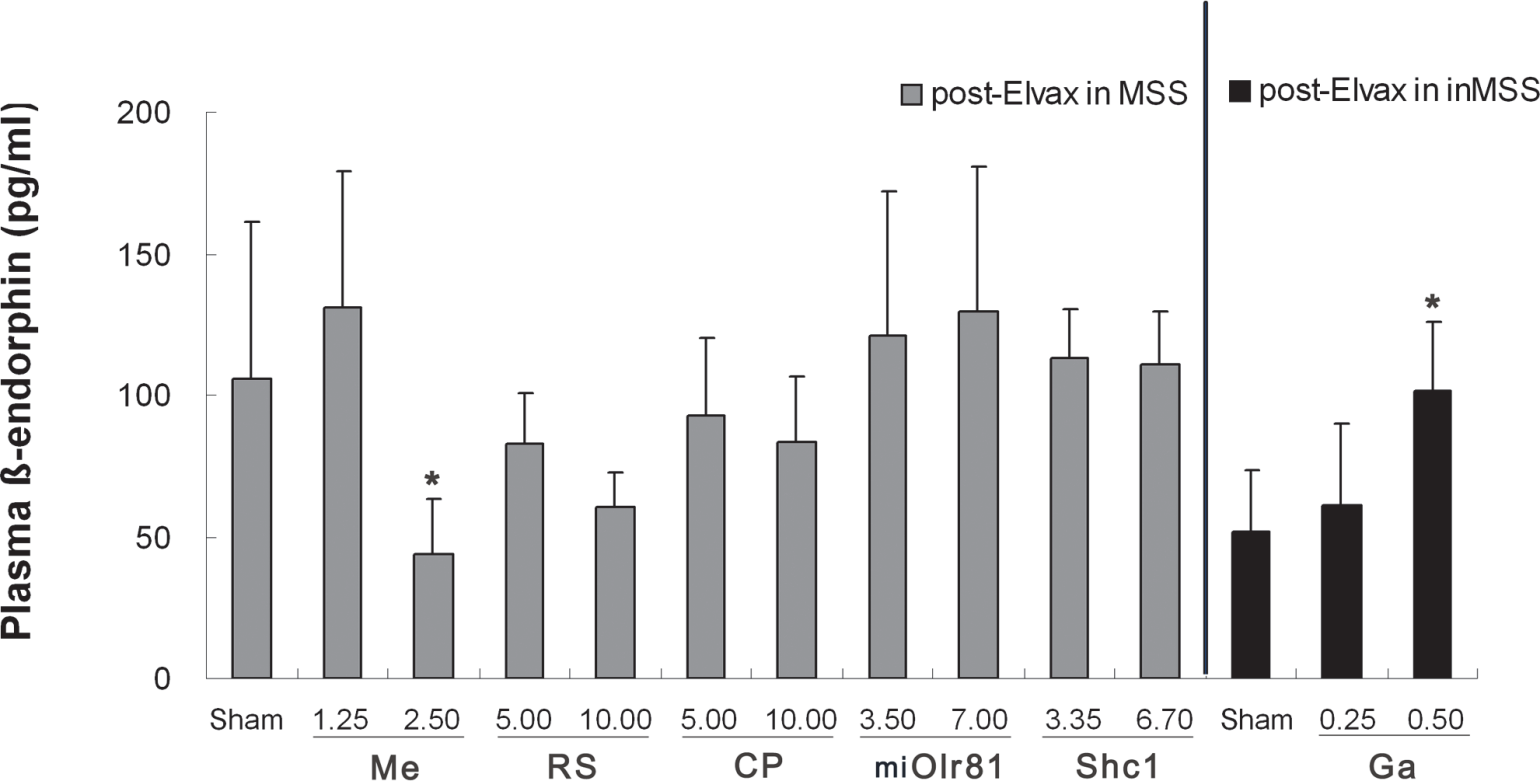
Effects of Elvax implantation over the CVN on plasma β-endorphin level after rotation stimulation. The final concentration of mecamylamine (Me), RS39604 (RS, CP99994 (CP) and gabazine (Ga) and the final titer of pAd-shOlr81 (shOlr81) and pAd-Shc1 (Shc1) in Elvax, and the number of animals used in each treatment group are the same as those in Fig 5. All data presented were expressed as the mean and vertical bars represent SEM. * P<0.05 compared with sham control group.

T-test analysis found that plasma β-endorphin levels were significantly increased in the MSS sham group compared to the inMSS sham group (t = 2.439, P = 0.030). One-way ANOVA analysis revealed a significant effect of mecamylamine-Elvax implantation (P = 0.044) which deceased plasma β-endorphin levels at 2.50 mM compared to sham operation MSS animals (P = 0.021), while RS39604 and CP99994 treatment had no effect on β-endorphin levels (P = 0.234, 0.596). Neither Olr81 knockdown nor Shc1 over-expression had an effect on plasma β-endorphin levels (P = 0.144, 0.949). In inMSS animals, gabazine induced a dose-dependent increase in plasma β-endorphin levels (P = 0.042) and a significant effect was shown at 5.0 mM when compared to sham operation inMSS animals (P = 0.021, Fig 7).

#### Summarization of Elvax implantation experiment

The results of the Elvax implantation experiment are summarized in Table 3. The nAchR inhibitor (mecamylamine) and adenovirus-mediated Olr81 interference improved both the defecation response and spontaneous locomotion activity in MSS animals, while the NK_1_R receptor inhibitor (CP99994) only alleviated the defecation response. nAchR inhibition also ameliorated the hormone response at high concentration. 5-HT_4_R inhibition (RS39604) and Shc1 over-expression had no beneficial effect on MS symptoms and stress hormone levels. 5-HT_4_R antagonism even aggravated the defecation response at high concentrations. In inMSS animals, GABA_A_R antagonism (Gabazine) decreased spontaneous locomotion activity and simultaneously enhanced the hormone response.

**Table 3:** Summary of Elvax-implantation effects on MSS indices.

	Defecation response	Distance traveled	Immobile duration	Center-point moving	β-endorphin
Mecamylamine	++	+	+	+	+
RS39604	-	-	-	-	-
CP99994	++	-	-	-	-
pAd-miOlr81	+	+	-	-	-
pAd-Shc1	-	-	-	+/-	-
Gabazine	+/-	++	+/-	++	+

++ effective at both concentrations/titers

+ effective only at higher concentration/titer

+/- insignificantly effective trend at higher concentrations/titers

- ineffective at both concentrations/titers

## Discussion

The reason for great individual differences in MS susceptibility are poorly understood and has received great attention in recent years.[2,39]. In humans, MS susceptibility is normally predicted by Motion Sickness Susceptibility Questionnaire scores or by measures of motion sickness tolerance using laboratory motion devices [40]. In mammals having emetic reflexes, such as dogs, cats, monkeys, and *Suncus murinus*, the latency to emesis or the amount of emetic episodes during provocative motion stimulation is used for MS susceptibility evaluation [23,41–43]. In rodents, which cannot vomit, MS can be indexed by pica, conditioned taste aversion (conditioned gaping), defecation and urination response, as well as reductions in body temperature and spontaneous locomotion, etc. [44–46]. Accumulating evidence suggested that pica may not be a sensitive assay of MS due to its delay in peaking following initiation of MS stimulation and its prolonged recovery after MS habituation [30,47]. In contrast, conditioned taste aversion and conditioned gaping are believed to be indicative of MS-associated nausea in rats [48,49]. However, whether these indices can be used to estimate MS susceptibility needs further investigation due to potential variability in chemical sensing and/or odor-laced context memory formation process during conditioning trials among animals [50,51]. Recent studies have set a fecal incontinence-based MS indiex, which was sensitive to emetic agents showed a great variability in rodents possibly due to fundamental individual diversity in MS susceptibility [47,52,53]. In this study, we showed that rotation induced defecation incontinence and hypoactivity was completely abolished by scopolamine treatment and bilateral labyrinthectomy. These results indicate the validity of our MS behavioral model in rodents using Ferris wheel like rotation stimulation which has been used for MS habituation assessment in our previous study [26,30].

Motion sickness susceptible and insusceptible animals were separated from the normal adult male population based on the severity of rotation-induced defecation and hypoactivity, which remained unshakable even after Elvax implantation surgery. As far as we know, this is the first time to establish simple and stable MS susceptibility evaluation criteria for rodents. Furthermore, in MSS animals, MS susceptibility declines with increase of age from postnatal day 30 to 150 in male rats (data not shown). This observartion is consistent with the fact that MS susceptibility reduces across ageing in humans [54]. However, for the fact that MS susceptibility fluctuates over the menstrual cycle in women, our model might not be effective and applicable for female animals [55]. In addition to age and sex, individual differences in vestibular function, which can be assessed by vestibular-ocular reflex dynamics, vestibular myogenic evoked potentials and postural activities, are believed to be the predictive factor specific to MS susceptibility [56]. In this study, difference in MS symptoms reflects the variability in vestibular induced autonomic reaction and depression like behavioral responses, indicating that our model for rodents could be used to investigate such vestibular related physiological processes and function in animals with different MS susceptibility. In addition, MSS animals exhibited a higher plasma levels of the endogenous opioid receptor agonist β-endorphin than inMSS animals after rotation exposure. Previous studies showed that plasma β-endorphin levels rose in response to MS and recovered to normal levels after repeated motion exposure in human subjects, while opioid receptor antagonism can increase MS susceptibility in human subjects and delay MS habituation in suncus murinus [57,58]. However, the value of β-endorphin as a plasma indicator for MS susceptibility still awaits further evaluation.

The current study also demonstrated a different gene expression pattern in the CVN between MSS-Rot and inMSS-Rot animals and the differential gene expression profile is completely different from those obtained in previous vestibular lesion studies [59,60]. Meanwhile, we found no MSS-Rot/inMSS-Rot expression difference in those genes that have been observed to be differentially expressed in the VN of motion-exposed animals compared with static controls (e.g., in protein level of c-fos, Trk receptors, calcitonin gene-related peptide) [26–29]. Hence, the differential gene expression profile that we identified might be exclusively related to the difference in functional alteration in CVN neurons between animals differing in MS susceptibility properties, but not to common responses induced by vestibular stimulation. These results provided the evidence that vestibular nucleus neurons of MSS and inMSS animals might have specific characteristics and molecular basis to re-achieve homeostasis after motion stimulation.

Electrophysiological experiments have demonstrated that the nAchR antagonist mecamylamine can block nicotine- or 1-dimethyl-4-phenylpiperazinium-induced membrane depolarization of MVN neurons and inhibit the α_3_-containing nAchR mediated presynaptic release of dopamine and noradrenaline [61–64]. Human studies showed that nicotine nasal spray can increase sensitivity to MS, while short-term smoking deprivation can enhance tolerance to MS [65,66]. These findings indicate that nAchR may have a direct regulatory role on the excitability of CVN neurons during MS. Furthermore, we found that MS susceptibility remarkably declined in MSS animals after receiving CVN administration of mecamylamine. With regard to clinical application, mecamylamine has several advantages over mAchR inhibitor scopolamine, which is the most widely used preventive drug for MS. Mecamylamine has a much longer duration of action than scopolamine (22 h versus 6 h) [67]. Recent studies showed that mecamylamine, at 3-fold lower doses than those used to treat hypertension (2.5–10 mg/day versus 30–90 mg/day), also showed significant central effects with much fewer and more manageable peripheral side effects [68]. Moreover, mecamylamine also has potential anti-addictive effects against methamphetamine abuse, which suggested that mecamylamine might be more appropriate than scopolamine to be used in combination with amphetamine to enhance the anti-MS effects [69,70]. These demonstrations suggest the possibility of using mecamylamine as potential therapeutic agents against MS in humans especially those extremely susceptible ones. On the other hand, our present study also showed that the GABA_A_R antagonist gabazine significantly enhanced MS susceptibility in inMSS animals. This result was inconsistent with a randomized, prospective, double-blind study which showed that oral administration of lorazepam (1 mg) cannot alleviate the simulated symptoms of space MS [71]. Given that the α_6_-containing receptor was diazepam-insensitive but gabazine is more potent at α6, than at α1, 2, and 3 subunit-containing receptors [72], we presume that the α_6_-containing GABA_A_R may be more prominent than other subtypes in regulating vestibular function especially in MS susceptible individual. In addtion, it has been reported that peripheral administration of CP99994 can suppress motion-induced vomiting in the cat, and inhibit hypergravity-induced pica in rats [73], but 5-HT_4_ receptor antagonism cannot reduce the emetic responses triggered by motion stimuli in *Suncus murinus* [74]. Here we showed that the NK_1_R blocker CP99994 ameliorated the defecation response, but not spontaneous locomotion or plasma hormone responses, while 5-HT_4_R antagonist RS39604 had no therapeutic effect on any of these indices. These results provided the evidence that nAchR, GABA_A_R, 5-HT_4_R and NK_1_R expressed in the CVN neurons contribute unequally to individual variability in MS susceptibility and may be involved in regulation of different vestibular mediated MS clinical manifestations. Nevertheless, our results failed to demonstrate the precise pathophysiology of hypoactivity symptoms observed in MSS animals. Some studies provided the evidences that connections between VN complex and motor-related area of the brain, such as the basal ganglia, may mediate some locomotor behavior responses of vestibular dysfunction [75]. Vestibular ascending pathways involved in fear and anxiety may contribute to increased immobility provoked by aversive motion stimuli [76], while reduced rearing was possibly due to vestibulospinal reflexes deficits and poor balance control [77]. Additional work is needed to clarify the neural pathways and molecular basis underlying vestibule-locomotor regulation.

Olfactory receptor is a category of GPCRs that mediate olfactory signaling via cAMP-dependent opening of calcium-permeable cyclic nucleotide-gated channels in olfactory sensory neurons [38,78]. Early in the 1980s, a neurohumoral hypothesis suggested that motion-induced vomiting might be evoked by some type of neurochemical agent in the cerebrospinal fluid (CSF) of the fourth ventricle [79,80]. Our current study found a large amount of OR genes differentially expressed between MSS-Rot and inMSS-Rot animals. We also showed that the basic mRNA level of Olr81 is higher in CVN of the MSS group than in inMSS group and Olr81 knock-down in the CVN significant alleviated the MS related symptoms. These results indicated that elevated OR gene expression levels might be related to relative high sensitivity to motion stimulation. However, whether OR activity is altered due to possible concentration fluctuations of some chemicals in the CSF of the fourth ventricle in MSS-Rot animals needs to be elucidated. Furthermore, we also found that Shc1 (p66Shc isoform), a key molecule in insulin receptors (IRs) signaling pathways, was down-regulated in inMSS-Rot animals, yet Shc1 over-expression did not alleviate the MS responses [81]. Our previous study showed that an intraperitoneal injection of insulin prior to motion exposure can alleviate MS symptoms in rats [43]. The contribution of the IRs/Shc1 pathway in CVN neurons to MS susceptibility warrants further investigation.

### Limitations

A limitation of this study is that only a limited number of animals were investigated and a larger sample size would have been preferable. Because the animals with extreme MS susceptibility differences were carefully screened to minimize the random error in MS symptoms within each MSS or inMSS group, the limited sample size appears to be less critical as the animals’ behavioral responses were easily distinguishable after pharmacologic treatment.

A potential disadvantage of Elvax technique may arise from possible surgery-induced anatomical lesion down to a depth approximately 100–300 μm underneath the tissue surface [82]. Considering that the depth of CVN from the brain stem surface is about 1 mm, such range of injury might be acceptable and this impact can be ruled out by setting sham operation control groups in our experiment design. Although assessment of release kinetics of adenovirus vectors were not performed in our study, the manipulation of candidate gene expression was successfully achieved and verified by RT-qPCR and western blot analysis. Meanwhile, it has been reported that freeze-drying process in ELVAX preparation could stabilize adenovirus for gene delivery systems [83]. These results support the notion that Elvax technique is effective for in situ delivery of inorganic compounds such as N-methyl-D-aspartate receptor antagonist, AchR antagonist, GABA receptor agonist [31,32], and is also efficient for controlled release of biomacromolecules including bovine serum albumin, antibody, enzymes and hormones in varieties of rat organs as well [31–33,82,84–86].

The present study did not examine the effect of tested drugs on sleep rhythm and motor coordination, which could have some impact on the results of behavioral evaluation for MS susceptibility. Although in situ drug administration can avoid most global effects on the bain, such possibilities must be highly addressed in our future pharmacological studies.

### Conclusions

There are apparent individual differences with respect to MS susceptibility in rats and that MSS and inMSS animals can be separated from the normal adult male population by assessing rotation-induced defecation and spontaneous locomotion in combination. There is a great difference in the CVN gene expression profile between MSS and inMSS animals exposed to motion stimulation and the differentially expressed genes contribute unequally to determine MS susceptibility. These findings highlight the link between active gene expression regulation in CVN neurons and variability in motion sickness susceptibility and provide potential targets for prevention or treatment of MS, especially for susceptible populations.

## Supporting Information

S1 Fig Verification of adenovirus delivery by Elvax implantation on expression of Olr81 and Shc1 in the CVN. (A) Statistical plot of data for RT-qPCR analysis of Olr81 and Shc1 mRNA levels. (B) Representative image (left) and statistical plot (right) of data for western blot analysis of Olr81 and Shc1 protein levels. The final titer of pAd-shOlr81 (shOlr81) and pAd-Shc1 (Shc1) in Elvax and the number of animals used in each group are the same as those in Fig 5. Values are expressed as the percentage of their corresponding GAPDH values and shown as the means (±S.E.). ** P<0.01, * P<0.05, compared with corresponding sham operation group.
(TIF)

S1 Table The primer sequences used for real-time PCR. (DOC)

S2 Table Fifty-five of the most up-regulated genes in the CVN of MSS-Rot animals compared with the inMSS-Rot group. (DOC)

S3 Table Seventeen of the most down-regulated genes in the CVN of MSS-Rot animals compared to the inMSS-Rot group. (DOC)

S4 Table The statistically significant up-regulated and down-regulated pathways (p<0.05) and the number of associated genes in each pathway. (DOC)

S5 Table Real-time qPCR verification of microarray data for differentially expressed genes in the CVN. (DOC)

## References

[1] Balaban CD (1999) Vestibular autonomic regulation (including motion sickness and the mechanism of vomiting). Curr Opin Neurol 12: 29–33. 10097881 10.1097/00019052-199902000-00005

[2] Shupak A, Gordon CR (2006) Motion sickness: advances in pathogenesis, prediction, prevention, and treatment. Aviat Space Environ Med 77: 1213–1223. 17183916

[3] Reason JT (1978) Motion sickness adaptation: a neural mismatch model. J R Soc Med 71: 819–829. 731645 10.1177/014107687807101109 PMC1436193

[4] Keshavarz B, Hettinger LJ, Kennedy RS, Campos JL (2014) Demonstrating the potential for dynamic auditory stimulation to contribute to motion sickness. PLoS One 9: e101016 10.1371/journal.pone.0101016 24983752 PMC4077751

[5] Stoffregen TA, Chen YC, Koslucher FC (2014) Motion control, motion sickness, and the postural dynamics of mobile devices. Exp Brain Res 232: 1389–1397. 10.1007/s00221-014-3859-3 24504199

[6] Stoffregen TA, Chen FC, Varlet M, Alcantara C, Bardy BG (2013) Getting Your Sea Legs. PLoS One 8: e66949 23840560 10.1371/journal.pone.0066949 PMC3686767

[7] Smart LJ Jr., Pagulayan RJ, Stoffregen TA (1998) Self-induced motion sickness in unperturbed stance. Brain Res Bull 47: 449–457. 10052573 10.1016/s0361-9230(98)00103-8

[8] Chouker A, Kaufmann I, Kreth S, Hauer D, Feuerecker M, Thieme D, et al (2010) Motion sickness, stress and the endocannabinoid system. PLoS One 5: e10752 10.1371/journal.pone.0010752 20505775 PMC2873996

[9] Johnson WH, Sunahara FA, Landolt JP (1999) Importance of the vestibular system in visually induced nausea and self-vection. J Vestib Res 9: 83–87. 10378179

[10] Ossenkopp KP, Parker LA, Limebeer CL, Burton P, Fudge MA, Cross-Mellor SK (2003) Vestibular lesions selectively abolish body rotation-induced, but not lithium-induced, conditioned taste aversions (oral rejection responses) in rats. Behav Neurosci 117: 105–112. 12619913

[11] Cullen KE (2012) The vestibular system: multimodal integration and encoding of self-motion for motor control. Trends Neurosci 35: 185–196. 10.1016/j.tins.2011.12.001 22245372 PMC4000483

[12] Beraneck M, Cullen KE (2007) Activity of vestibular nuclei neurons during vestibular and optokinetic stimulation in the alert mouse. J Neurophysiol 98: 1549–1565. 17625061 10.1152/jn.00590.2007

[13] Sadeghi SG, Mitchell DE, Cullen KE (2009) Different neural strategies for multimodal integration: comparison of two macaque monkey species. Exp Brain Res 195: 45–57. 10.1007/s00221-009-1751-3 19283371 PMC3319768

[14] Aleksandrov VG, Bagaev VA, Nozdrachev AD (1998) Gastric related neurons in the rat medial vestibular nucleus. Neurosci Lett 250: 66–68. 9696067 10.1016/s0304-3940(98)00408-x

[15] Miller DM, Cotter LA, Gandhi NJ, Schor RH, Cass SP, Huff NO, et al (2008) Responses of caudal vestibular nucleus neurons of conscious cats to rotations in vertical planes, before and after a bilateral vestibular neurectomy. Exp Brain Res 188: 175–186. 10.1007/s00221-008-1359-z 18368395 PMC2440585

[16] Balaban CD, Beryozkin G (1994) Vestibular nucleus projections to nucleus tractus solitarius and the dorsal motor nucleus of the vagus nerve: potential substrates for vestibulo-autonomic interactions. Exp Brain Res 98: 200–212. 8050507 10.1007/BF00228409

[17] Balaban CD (1996) Vestibular nucleus projections to the parabrachial nucleus in rabbits: implications for vestibular influences on the autonomic nervous system. Exp Brain Res 108: 367–381. 8801117 10.1007/BF00227260

[18] Mori RL, Cotter LA, Arendt HE, Olsheski CJ, Yates BJ (2005) Effects of bilateral vestibular nucleus lesions on cardiovascular regulation in conscious cats. J Appl Physiol (1985) 98: 526–533. 15475594 10.1152/japplphysiol.00970.2004

[19] Lai CH, Tse YC, Shum DK, Yung KK, Chan YS (2004) Fos expression in otolith-related brainstem neurons of postnatal rats following off-vertical axis rotation. J Comp Neurol 470: 282–296. 14755517 10.1002/cne.11048

[20] Pompeiano O, d'Ascanio P, Balaban E, Centini C, Pompeiano M (2004) Gene expression in autonomic areas of the medulla and the central nucleus of the amygdala in rats during and after space flight. Neuroscience 124: 53–69. 14960339 10.1016/j.neuroscience.2003.09.027

[21] Zhang L, Mao JF, Wu XN, Bao YC (2014) [A randomized controlled trial: acclimatization training on the prevention of motion sickness in hot-humid environment]. Zhongguo Ying Yong Sheng Li Xue Za Zhi 30: 279–284. 25244801

[22] Chan YS, Cheung YM (1992) Response of otolith-related neurons in bilateral vestibular nucleus of acute hemilabyrinthectomized cats to off-vertical axis rotations. Ann N Y Acad Sci 656: 755–765. 1599181 10.1111/j.1749-6632.1992.tb25254.x

[23] Balaban CD, Ogburn SW, Warshafsky SG, Ahmed A, Yates BJ (2014) Identification of neural networks that contribute to motion sickness through principal components analysis of fos labeling induced by galvanic vestibular stimulation. PLoS One 9: e86730 10.1371/journal.pone.0086730 24466215 PMC3900607

[24] Markia B, Kovacs ZI, Palkovits M (2008) Projections from the vestibular nuclei to the hypothalamic paraventricular nucleus: morphological evidence for the existence of a vestibular stress pathway in the rat brain. Brain Struct Funct 213: 239–245. 10.1007/s00429-008-0172-6 18247051

[25] Arshian MS, Puterbaugh SR, Miller DJ, Catanzaro MF, Hobson CE, McCall AA, et al (2013) Effects of visceral inputs on the processing of labyrinthine signals by the inferior and caudal medial vestibular nuclei: ramifications for the production of motion sickness. Exp Brain Res 228: 353–363. 10.1007/s00221-013-3568-3 23712685 PMC3706452

[26] Cai YL, Wang JQ, Chen XM, Li HX, Li M, Guo JS (2010) Decreased Fos protein expression in rat caudal vestibular nucleus is associated with motion sickness habituation. Neurosci Lett 480: 87–91. 10.1016/j.neulet.2010.06.011 20540989

[27] Xiaocheng W, Zhaohui S, Junhui X, Lei Z, Lining F, Zuoming Z (2012) Expression of calcitonin gene-related peptide in efferent vestibular system and vestibular nucleus in rats with motion sickness. PLoS One 7: e47308 10.1371/journal.pone.0047308 23056625 PMC3467246

[28] Zhang FX, Lai CH, Tse YC, Shum DK, Chan YS (2005) Expression of Trk receptors in otolith-related neurons in the vestibular nucleus of rats. Brain Res 1062: 92–100. 16256078 10.1016/j.brainres.2005.09.025

[29] Xiaocheng W, Zhaohui S, Ka B, Junhui X, Lei Z, Feng X, et al (2013) The expression of calcitonin gene-related Peptide and acetylcholine in the vestibular-related nucleus population of wild-type mice and retinal degeneration fast mice after rotary stimulation. J Mol Neurosci 51: 514–521. 10.1007/s12031-013-0087-4 24037277

[30] Wang JQ, Li HX, Chen XM, Mo FF, Qi RR, Guo JS, et al (2012) Temporal change in NMDA receptor signaling and GABAA receptor expression in rat caudal vestibular nucleus during motion sickness habituation. Brain Res 1461: 30–40. 10.1016/j.brainres.2012.04.041 22608069

[31] Tu S, Butt CM, Pauly JR, Debski EA (2000) Activity-dependent regulation of substance P expression and topographic map maintenance by a cholinergic pathway. J Neurosci 20: 5346–5357. 10884319 10.1523/JNEUROSCI.20-14-05346.2000 PMC2265086

[32] Nodal FR, Bajo VM, King AJ (2012) Plasticity of spatial hearing: behavioural effects of cortical inactivation. J Physiol 590: 3965–3986. 10.1113/jphysiol.2011.222828 22547635 PMC3464400

[33] Anomal R, de Villers-Sidani E, Merzenich MM, Panizzutti R (2013) Manipulation of BDNF signaling modifies the experience-dependent plasticity induced by pure tone exposure during the critical period in the primary auditory cortex. PLoS One 8: e64208 10.1371/journal.pone.0064208 23700463 PMC3660256

[34] Kilkenny C, Browne WJ, Cuthi I, Emerson M, Altman DG (2012) Improving bioscience research reporting: the ARRIVE guidelines for reporting animal research. Vet Clin Pathol 41: 27–31. 10.1111/j.1939-165X.2012.00418.x 22390425

[35] McGrath JC, Drummond GB, McLachlan EM, Kilkenny C, Wainwright CL (2010) Guidelines for reporting experiments involving animals: the ARRIVE guidelines. Br J Pharmacol 160: 1573–1576. 10.1111/j.1476-5381.2010.00873.x 20649560 PMC2936829

[36] Cai YL, Ma WL, Li M, Guo JS, Li YQ, Wang LG, et al (2007) Glutamatergic vestibular neurons express Fos after vestibular stimulation and project to the NTS and the PBN in rats. Neurosci Lett 417: 132–137. 17412503 10.1016/j.neulet.2007.01.079

[37] Glusman G, Bahar A, Sharon D, Pilpel Y, White J, Lancet D (2000) The olfactory receptor gene superfamily: data mining, classification, and nomenclature. Mamm Genome 11: 1016–1023. 11063259 10.1007/s003350010196

[38] Niimura Y (2012) Olfactory receptor multigene family in vertebrates: from the viewpoint of evolutionary genomics. Curr Genomics 13: 103–114. 23024602 10.2174/138920212799860706 PMC3308321

[39] Schmal F (2013) Neuronal mechanisms and the treatment of motion sickness. Pharmacology 91: 229–241. 10.1159/000350185 23615033

[40] Golding JF (1998) Motion sickness susceptibility questionnaire revised and its relationship to other forms of sickness. Brain Res Bull 47: 507–516. 10052582 10.1016/s0361-9230(98)00091-4

[41] Conder GA, Sedlacek HS, Boucher JF, Clemence RG (2008) Efficacy and safety of maropitant, a selective neurokinin 1 receptor antagonist, in two randomized clinical trials for prevention of vomiting due to motion sickness in dogs. J Vet Pharmacol Ther 31: 528–532. 10.1111/j.1365-2885.2008.00990.x 19000275

[42] Javid FA, Naylor RJ (2002) The effect of serotonin and serotonin receptor antagonists on motion sickness in Suncus murinus. Pharmacol Biochem Behav 73: 979–989. 12213545 10.1016/s0091-3057(02)00955-3

[43] Mo FF, Qin HH, Wang XL, Shen ZL, Xu Z, Wang KH, et al (2012) Acute hyperglycemia is related to gastrointestinal symptoms in motion sickness: an experimental study. Physiol Behav 105: 394–401. 10.1016/j.physbeh.2011.08.024 21907224

[44] Ossenkopp KP, Rabi YJ, Eckel LA, Hargreaves EL (1994) Reductions in body temperature and spontaneous activity in rats exposed to horizontal rotation: abolition following chemical labyrinthectomy. Physiol Behav 56: 319–324. 7938244 10.1016/0031-9384(94)90201-1

[45] McCaffrey RJ (1985) Appropriateness of kaolin consumption as an index of motion sickness in the rat. Physiol Behav 35: 151–156. 4070378 10.1016/0031-9384(85)90329-4

[46] Ossenkopp KP, Frisken NL (1982) Defecation as an index of motion sickness in the rat. Physiol Psychol 10: 355–360.

[47] Yu XH, Cai GJ, Liu AJ, Chu ZX, Su DF (2007) A novel animal model for motion sickness and its first application in rodents. Physiol Behav 92: 702–707. 17612582 10.1016/j.physbeh.2007.05.067

[48] Limebeer CL, Krohn JP, Cross-Mellor S, Litt DE, Ossenkopp KP, Parker LA (2008) Exposure to a context previously associated with nausea elicits conditioned gaping in rats: a model of anticipatory nausea. Behav Brain Res 187: 33–40. 17897732 10.1016/j.bbr.2007.08.024

[49] Cordick N, Parker LA, Ossenkopp KP (1999) Rotation-induced conditioned rejection in the taste reactivity test. Neuroreport 10: 1557–1559. 10380980 10.1097/00001756-199905140-00030

[50] Kraemer S, Apfelbach R (2004) Olfactory sensitivity, learning and cognition in young adult and aged male Wistar rats. Physiol Behav 81: 435–442. 15135015 10.1016/j.physbeh.2004.01.012

[51] Rahn EJ, Guzman-Karlsson MC, David Sweatt J (2013) Cellular, molecular, and epigenetic mechanisms in non-associative conditioning: implications for pain and memory. Neurobiol Learn Mem 105: 133–150. 10.1016/j.nlm.2013.06.008 23796633 PMC3769437

[52] Wei X, Wang ZB, Zhang LC, Liu WY, Su DF, Li L (2011) Verification of motion sickness index in mice. CNS Neurosci Ther 17: 790–792. 10.1111/j.1755-5949.2011.00272.x 22117804 PMC6493908

[53] Horn CC, Kimball BA, Wang H, Kaus J, Dienel S, Nagy A, et al (2013) Why can't rodents vomit? A comparative behavioral, anatomical, and physiological study. PLoS One 8: e60537 10.1371/journal.pone.0060537 23593236 PMC3622671

[54] Paillard AC, Quarck G, Paolino F, Denise P, Paolino M, Golding JF, et al (2013) Motion sickness susceptibility in healthy subjects and vestibular patients: effects of gender, age and trait-anxiety. J Vestib Res 23: 203–209. 10.3233/VES-130501 24284600

[55] Golding JF, Kadzere P, Gresty MA (2005) Motion sickness susceptibility fluctuates through the menstrual cycle. Aviat Space Environ Med 76: 970–973. 16235881

[56] Golding JF (2006) Motion sickness susceptibility. Auton Neurosci 129: 67–76. 16931173 10.1016/j.autneu.2006.07.019

[57] Javid FA, Naylor RJ (2001) Opioid receptor involvement in the adaptation to motion sickness in Suncus murinus. Pharmacol Biochem Behav 68: 761–767. 11526974 10.1016/s0091-3057(01)00470-1

[58] Kohl RL (1992) beta-Endorphin and arginine vasopressin following stressful sensory stimuli in man. Aviat Space Environ Med 63: 986–993. 1332670

[59] Horii A, Masumura C, Smith PF, Darlington CL, Kitahara T, Uno A, et al (2004) Microarray analysis of gene expression in the rat vestibular nucleus complex following unilateral vestibular deafferentation. J Neurochem 91: 975–982. 15525351 10.1111/j.1471-4159.2004.02781.x

[60] Park MK, Lee BD, Lee JD, Jung HH, Chae SW (2012) Gene profiles during vestibular compensation in rats after unilateral labyrinthectomy. Ann Otol Rhinol Laryngol 121: 761–769. 23193910 10.1177/000348941212101110

[61] Improgo MR, Scofield MD, Tapper AR, Gardner PD (2010) The nicotinic acetylcholine receptor CHRNA5/A3/B4 gene cluster: dual role in nicotine addiction and lung cancer. Prog Neurobiol 92: 212–226. 10.1016/j.pneurobio.2010.05.003 20685379 PMC2939268

[62] Morley BJ (1997) The embryonic and post-natal expression of the nicotinic receptor alpha 3-subunit in rat lower brainstem. Brain Res Mol Brain Res 48: 407–412. 9332738 10.1016/s0169-328x(97)00159-9

[63] Phelan KD, Gallagher JP (1992) Direct muscarinic and nicotinic receptor-mediated excitation of rat medial vestibular nucleus neurons in vitro. Synapse 10: 349–358. 1585263 10.1002/syn.890100410

[64] Gotti C, Zoli M, Clementi F (2006) Brain nicotinic acetylcholine receptors: native subtypes and their relevance. Trends Pharmacol Sci 27: 482–491. 16876883 10.1016/j.tips.2006.07.004

[65] Zingler VC, Denecke K, Jahn K, von Meyer L, Krafczyk S, Krams M, et al (2007) The effect of nicotine on perceptual, ocular motor, postural, and vegetative functions at rest and in motion. J Neurol 254: 1689–1697. 17990061 10.1007/s00415-007-0621-9

[66] Golding JF, Prosyanikova O, Flynn M, Gresty MA (2011) The effect of smoking nicotine tobacco versus smoking deprivation on motion sickness. Auton Neurosci 160: 53–58. 10.1016/j.autneu.2010.09.009 21036110

[67] Nickell JR, Grinevich VP, Siripurapu KB, Smith AM, Dwoskin LP (2013) Potential therapeutic uses of mecamylamine and its stereoisomers. Pharmacol Biochem Behav 108: 28–43. 10.1016/j.pbb.2013.04.005 23603417 PMC3690754

[68] Shytle RD, Penny E, Silver AA, Goldman J, Sanberg PR (2002) Mecamylamine (Inversine): an old antihypertensive with new research directions. J Hum Hypertens 16: 453–457. 12080428 10.1038/sj.jhh.1001416

[69] Glick SD, Sell EM, Maisonneuve IM (2008) Brain regions mediating alpha3beta4 nicotinic antagonist effects of 18-MC on methamphetamine and sucrose self-administration. Eur J Pharmacol 599: 91–95. 10.1016/j.ejphar.2008.09.038 18930043 PMC2600595

[70] Takeda N, Morita M, Yamatodani A, Wada H, Matsunaga T (1990) Catecholaminergic responses to rotational stress in rat brain stem: implications for amphetamine therapy of motion sickness. Aviat Space Environ Med 61: 1018–1021. 2256874

[71] Dornhoffer J, Chelonis JJ, Blake D (2004) Stimulation of the semicircular canals via the rotary chair as a means to test pharmacologic countermeasures for space motion sickness. Otol Neurotol 25: 740–745. 15354005 10.1097/00129492-200409000-00016

[72] Olsen RW, Sieghart W (2008) International Union of Pharmacology. LXX. Subtypes of gamma-aminobutyric acid(A) receptors: classification on the basis of subunit composition, pharmacology, and function. Update. Pharmacol Rev 60: 243–260. 10.1124/pr.108.00505 18790874 PMC2847512

[73] Lucot JB, Obach RS, McLean S, Watson JW (1997) The effect of CP-99994 on the responses to provocative motion in the cat. Br J Pharmacol 120: 116–120. 9117085 10.1038/sj.bjp.0700888 PMC1564356

[74] Nakayama H, Yamakuni H, Higaki M, Ishikawa H, Imazumi K, Matsuo M, et al (2005) Antiemetic activity of FK1052, a 5-HT3- and 5-HT4-receptor antagonist, in Suncus murinus and ferrets. J Pharmacol Sci 98: 396–403. 16079468 10.1254/jphs.fpj05001x

[75] Stiles L, Smith PF (2014) The vestibular—basal ganglia connection: Balancing motor control. Brain Res. 10.1016/j.brainres.2014.11.063 25498858

[76] Coelho CM, Balaban CD (2015) Visuo-vestibular contributions to anxiety and fear. Neurosci Biobehav Rev 48C: 148–159. 10.1016/j.neubiorev.2014.10.023 25451199

[77] Goddard M, Zheng Y, Darlington CL, Smith PF (2008) Locomotor and exploratory behavior in the rat following bilateral vestibular deafferentation. Behav Neurosci 122: 448–459. 10.1037/0735-7044.122.2.448 18410183

[78] Kato A, Touhara K (2009) Mammalian olfactory receptors: pharmacology, G protein coupling and desensitization. Cell Mol Life Sci 66: 3743–3753. 10.1007/s00018-009-0111-6 19652915 PMC11115879

[79] Lucot JB, Crampton GH, Matson WR, Gamache PH (1989) Cerebrospinal fluid constituents of cat vary with susceptibility to motion sickness. Life Sci 44: 1239–1245. 2469925 10.1016/0024-3205(89)90359-7

[80] Contrucci RB, Wilpizeski CR (1985) Neurohumoral hypothesis of motion-induced vomiting. Communication. Ann Otol Rhinol Laryngol 94: 322–323. 2861784

[81] Berry A, Cirulli F (2013) The p66(Shc) gene paves the way for healthspan: evolutionary and mechanistic perspectives. Neurosci Biobehav Rev 37: 790–802. 10.1016/j.neubiorev.2013.03.005 23524280

[82] Persico AM, Calia E, Keller F (1997) Implants for sustained drug release over the somatosensory cortex of the newborn rat: a comparison of materials and surgical procedures. J Neurosci Methods 76: 105–113. 9334945 10.1016/s0165-0270(97)00087-3

[83] Talsma H, Cherng J, Lehrmann H, Kursa M, Ogris M, Hennink WE, et al (1997) Stabilization of gene delivery systems by freeze-drying. Int J Pharm 157: 233–238. 10477820 10.1016/s0378-5173(97)00244-5

[84] Langer R, Brem H, Tapper D (1981) Biocompatibility of polymeric delivery systems for macromolecules. J Biomed Mater Res 15: 267–277. 7348718 10.1002/jbm.820150212

[85] Silberstein GB, Daniel CW (1982) Elvax 40P implants: sustained, local release of bioactive molecules influencing mammary ductal development. Dev Biol 93: 272–278. 7128936 10.1016/0012-1606(82)90259-7

[86] Sefton MV, Brown LR, Langer RS (1984) Ethylene-vinyl acetate copolymer microspheres for controlled release of macromolecules. J Pharm Sci 73: 1859–1861. 6527279 10.1002/jps.2600731258

