# Supplementary figures and images for: Differential Gene Expression Profile in the Rat Caudal Vestibular Nucleus is Associated with Individual Differences in Motion Sickness Susceptibility
Source: PLoS One. 2015 Apr 24;10(4):e0124203. doi: 10.1371/journal.pone.0124203 (PMC4409317; doi:10.1371/journal.pone.0124203)

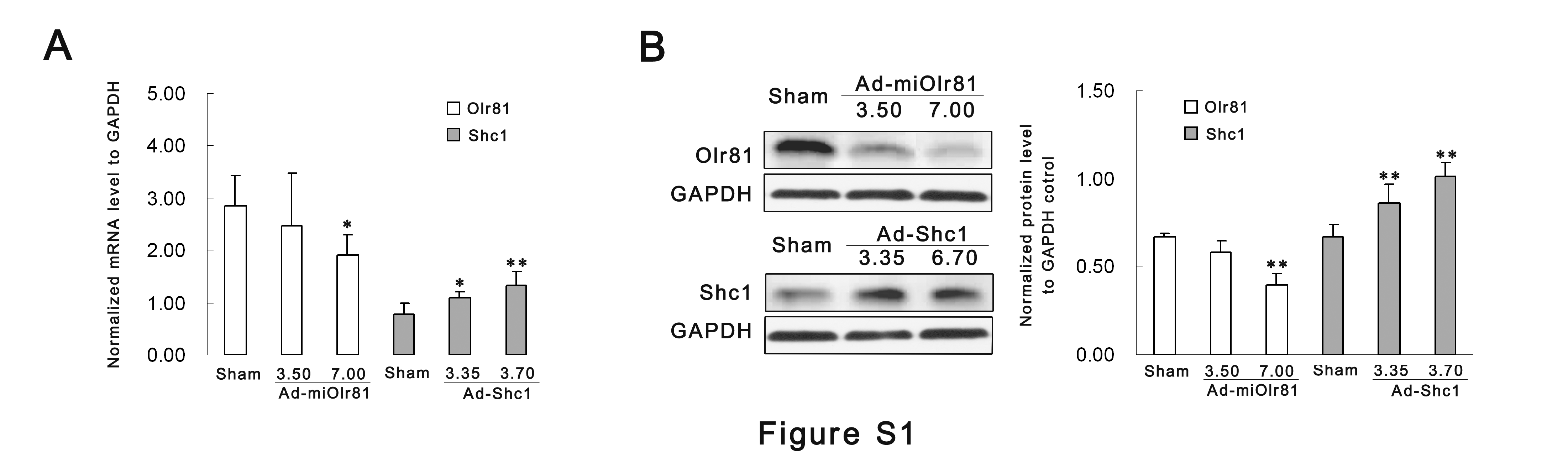

Supplement: S1 Fig — (A) Statistical plot of data for RT-qPCR analysis of Olr81 and Shc1 mRNA levels. (B) Representative image (left) and statistical plot (right) of data for western blot analysis of Olr81 and Shc1 protein levels. The final titer of pAd-shOlr81 (shOlr81) and pAd-Shc1 (Shc1) in Elvax and the number of animals used in each group are the same as those in Fig 5. Values are expressed as the percentage of their corresponding GAPDH values and shown as the means (±S.E.). ** P<0.01, * P<0.05, compared with corresponding sham operation group. (TIF) [file pone.0124203.s001.tif]
