# Supplementary material for: Differential Gene Expression Profile in the Rat Caudal Vestibular Nucleus is Associated with Individual Differences in Motion Sickness Susceptibility
Source: PLoS One. 2015 Apr 24;10(4):e0124203. doi: 10.1371/journal.pone.0124203 (PMC4409317; doi:10.1371/journal.pone.0124203)
Supplement: S1 Table — (DOC) [file pone.0124203.s002.doc]

**Table S1 The primer sequences used for real-time PCR.**

| gene | primer | |
| --- | --- | --- |
| Chrna3 | sense | 5’-TCATCCGGCCAGTGGCTAATGT-3’ |
|  | antisense | 5’-TGGAAATCCCCATCAGCGTTGT-3’ |
| Htr4 | sense | 5’-CAGGGACAGGCAGCTCAGGAAAAT-3’ |
|  | antisense | 5’-ATGCGATGCGTAGAGGGGTCA-3’ |
| Tacr1 | sense | 5’-TGCCTCAACGACAGGTTCCGT-3’ |
|  | antisense | 5’-TTGCTGCGAGAGGAGCCGTT-3’ |
| Gabra6 | sense | 5’-AGCCCCCGGTAGCAAAGTCA-3’ |
|  | antisense | 5’-GCCGCCAATGGCTGGTAAGA-3’ |
| Olr81 | sense | 5’-AACTTTGGTGGCTGTGTGGCTC-3’ |
|  | antisense | 5’-CCACAGGACAGTTTGGCAATGCT-3’ |
| Shc1 | sense | 5’-TTGCGTGGTCCGGAACCAGAT-3’ |
|  | antisense | 5’-CCACGCAACCCATGTACCGAA-3’ |
| GAPDH | sense | 5’-GGCTCTCTGCTCCTCCCTGTTCTA-3’ |
|  | antisense | 5’-CGTCCGATACGGCCAAATCCGT-3’ |
