# Supplementary material for: Differential Gene Expression Profile in the Rat Caudal Vestibular Nucleus is Associated with Individual Differences in Motion Sickness Susceptibility
Source: PLoS One. 2015 Apr 24;10(4):e0124203. doi: 10.1371/journal.pone.0124203 (PMC4409317; doi:10.1371/journal.pone.0124203)
Supplement: S2 Table — (DOC) [file pone.0124203.s003.doc]

**Table S2. Fifty-five of the most upregulated genes in the CVN of MSS-Rot animals compared with the inMSS-Rot group.**

| **Gene symbol** | **Description** | **p-value** | **MSS-Rot/inMSS-Rot** | **GO (Molecular function)** |
| --- | --- | --- | --- | --- |
| Crygf | crystallin, gamma F | 0.044598 | 2.13 | structural constituent of eye lens |
| Olr81 | olfactory receptor 81 | 0.003579 | 2.11 | receptor activity, olfactory receptor activity |
| Olr1022 | olfactory receptor 1022 | 0.041294 | 2.06 | receptor activity, olfactory receptor activity |
| Olr175 | olfactory receptor 175 | 0.013896 | 1.99 | receptor activity, olfactory receptor activity |
| Olr1147 | olfactory receptor 1147 | 0.02889 | 1.97 | receptor activity, olfactory receptor activity |
| Olr554 | olfactory receptor 554 | 0.015552 | 1.87 | receptor activity, olfactory receptor activity |
| Olr403 | olfactory receptor 403 | 0.010598 | 1.83 | receptor activity, olfactory receptor activity |
| Fcgr2a | Fc fragment of IgG, low affinity IIa, receptor | 0.01656 | 1.75 | receptor activity, protein binding |
| Oxgr1 | oxoglutarate (alpha-ketoglutarate) receptor 1 | 0.047825 | 1.67 | receptor activity, G-protein coupled receptor activity,purinergic nucleotide receptor activity |
| Olr841 | olfactory receptor 841 | 0.030514 | 1.67 | receptor activity, olfactory receptor activity |
| Spink1 | serine peptidase inhibitor, Kazal type 1 | 0.006056 | 1.66 | serine-type endopeptidase inhibitor activity, calmodulin binding,peptidase inhibitor activity |
| Olr1262 | olfactory receptor 1262 | 0.048272 | 1.66 | receptor activity, olfactory receptor activity |
| Olr1657 | olfactory receptor 1657 | 0.02129 | 1.63 | receptor activity, olfactory receptor activity |
| Olr241 | olfactory receptor 241 | 0.025197 | 1.61 | receptor activity, olfactory receptor activity |
| Vom1r23 | vomeronasal 1 receptor, 23 | 0.021578 | 1.59 | receptor activity, pheromone receptor activity |
| Dnajb12 | DnaJ (Hsp40) homolog, subfamily B, member 12 | 0.030966 | 1.58 | heat shock protein binding,unfolded protein binding |
| Olr766 | olfactory receptor 766 | 0.030408 | 1.56 | receptor activity, olfactory receptor activity |
| Acsbg2 | acyl-CoA synthetase bubblegum family member 2 | 0.013603 | 1.56 | nucleotide binding, long-chain fatty acid-CoA ligase activity |
| Olr1128 | olfactory receptor 1128 | 0.004119 | 1.55 | receptor activity, olfactory receptor activity |
| Olr1072 | olfactory receptor 1072 | 0.04292 | 1.51 | receptor activity, olfactory receptor activity |
| Ccl17 | chemokine (C-C motif) ligand 17 | 0.019019 | 1.50 | chemokine activity |
| Olr820 | olfactory receptor 820 | 0.029685 | 1.49 | receptor activity, olfactory receptor activity |
| Olr1297 | olfactory receptor 1297 | 0.039059 | 1.49 | receptor activity, olfactory receptor activity |
| Olr96 | olfactory receptor 96 | 0.032638 | 1.47 | receptor activity, olfactory receptor activity |
| Olr82 | olfactory receptor 82 | 0.023287 | 1.47 | receptor activity, olfactory receptor activity |
| S100g | S100 calcium binding protein G | 0.033061 | 1.46 | vitamin D binding, calcium ion binding |
| Ly49si1 | immunoreceptor Ly49si1 | 0.000525 | 1.46 | receptor activity, binding |
| Olr697 | olfactory receptor 697 | 0.001988 | 1.44 | receptor activity, olfactory receptor activity |
| Olr488 | olfactory receptor 488 | 0.006675 | 1.43 | receptor activity, olfactory receptor activity |
| Olr698 | olfactory receptor 698 | 0.044863 | 1.41 | receptor activity, olfactory receptor activity |
| Olr1073 | olfactory receptor 1073 | 0.048713 | 1.41 | receptor activity, olfactory receptor activity |
| Vom1r41 | vomeronasal 1 receptor, 41 | 0.003273 | 1.40 | receptor activity, pheromone receptor activity |
| Olr455 | olfactory receptor 455 | 0.017926 | 1.40 | receptor activity, olfactory receptor activity |
| Olr442 | olfactory receptor 442 | 0.010832 | 1.40 | receptor activity, olfactory receptor activity |
| Olr32 | olfactory receptor 32 | 0.022066 | 1.38 | receptor activity, olfactory receptor activity |
| Olr1475 | olfactory receptor 1475 | 0.001891 | 1.38 | receptor activity, olfactory receptor activity |
| Olr414 | olfactory receptor 414 | 0.041962 | 1.37 | receptor activity, olfactory receptor activity |
| Olr1536 | olfactory receptor 1536 | 0.014107 | 1.37 | receptor activity, olfactory receptor activity |
| Olr1375 | olfactory receptor 1375 | 0.004749 | 1.36 | receptor activity, olfactory receptor activity |
| Vom1r24 | vomeronasal 1 receptor, 24 | 0.043536 | 1.35 | receptor activity, pheromone receptor activity |
| Prp2 | proline rich protein 2 | 0.043834 | 1.35 | --- |
| Orm1 | orosomucoid 1 | 0.009372 | 1.35 | protein binding, drug binding |
| Olr545 | olfactory receptor 545 | 0.019495 | 1.35 | receptor activity, olfactory receptor activity |
| Olr223 | olfactory receptor 223 | 0.022278 | 1.35 | receptor activity, olfactory receptor activity |
| Olr1529 | olfactory receptor 1529 | 0.038392 | 1.35 | receptor activity, olfactory receptor activity |
