# Supplementary material for: Differential Gene Expression Profile in the Rat Caudal Vestibular Nucleus is Associated with Individual Differences in Motion Sickness Susceptibility
Source: PLoS One. 2015 Apr 24;10(4):e0124203. doi: 10.1371/journal.pone.0124203 (PMC4409317; doi:10.1371/journal.pone.0124203)
Supplement: S3 Table — (DOC) [file pone.0124203.s004.doc]

**Table S3. Seventeen of the most down-regulated genes in the CVN of MSS-Rot animals compared to the inMSS-Rot group.**

| **Gene symbol** | **Description** | **p-value** | **MSS-Rot/inMSS-Rot** | **GO (Molecular function)** |
| --- | --- | --- | --- | --- |
| Gabra6 | gamma-aminobutyric acid (GABA) A receptor, alpha 6 | 0.021189 | 0.56 | receptor activity, GABA-A receptor activity |
| Obp2b | odorant binding protein 2B | 0.034578 | 0.61 | transporter activity, binding |
| Prkacb | protein kinase, cAMP dependent, catalytic, beta | 0.001988 | 0.69 | protein amino acid phosphorylation |
| Olr1087 | olfactory receptor 1087 | 0.04268 | 0.69 | receptor activity, olfactory receptor activity |
| Nupr1 | nuclear protein, transcriptional regulator, 1 | 0.040287 | 0.69 | --- |
| Ube2d4 | ubiquitin-conjugating enzyme E2D 4 | 0.022306 | 0.71 | nucleotide binding, ubiquitin-protein ligase activity |
| Rrs1 | RRS1 ribosome biogenesis regulator homolog | 0.023235 | 0.71 | no biological data available |
| Klk1c10 | T-kininogenase | 0.044119 | 0.71 | catalytic activity, serine-type endopeptidase activity |
| Shc1 | SHC (Src homology 2 domain containing) transforming protein 1 (Shc1), transcript variant 2 | 0.0366554 | 0.71 | activation of MAPK activity |
| Olr1163 | olfactory receptor 1163 | 0.017454 | 0.72 | receptor activity, olfactory receptor activity |
| Olr250 | olfactory receptor 250 | 0.033403 | 0.73 | receptor activity, olfactory receptor activity |
| Rnf213 | ring finger protein 213 gene | 0.039859 | 0.73 | nucleotide binding,protein binding,zinc ion binding |
| Slc2a5 | solute carrier family 2, member 5 | 0.011784 | 0.74 | transporter activity, fructose transmembrane transporter activity, fructose transmembrane transporter |
| Slc9a3r1 | solute carrier family 9, member 3 regulator 1 | 0.048074 | 0.74 | protein binding, PDZ domain binding |
| Ctss | cathepsin S | 0.045959 | 0.74 | cysteine-type endopeptidase activity, peptidase activity |
| Igsf1 | immunoglobulin superfamily, member 1 | 0.038837 | 0.75 | protein binding, coreceptor |
| Stom | stomatin | 0.028773 | 0.75 | protein binding |
