# Supplementary material for: Differential Gene Expression Profile in the Rat Caudal Vestibular Nucleus is Associated with Individual Differences in Motion Sickness Susceptibility
Source: PLoS One. 2015 Apr 24;10(4):e0124203. doi: 10.1371/journal.pone.0124203 (PMC4409317; doi:10.1371/journal.pone.0124203)
Supplement: S4 Table — (DOC) [file pone.0124203.s005.doc]

|  | Pathway id | Pathway name | Gene count | P value |
| --- | --- | --- | --- | --- |
| up-regulated pathway | 4740 | Olfactory transduction | 51 | 3.52E-43 |
|  | 5332 | Graft-versus-host disease | 3 | 0.00636578 |
|  | 561 | Glycerolipid metabolism | 2 | 0.02672836 |
| down-regulated  pathway | 4910 | Insulin signaling pathway | 3 | 0.00938387 |
|  | 4742 | Taste transduction | 2 | 0.00970767 |
|  | 4310 | Wnt signaling pathway | 3 | 0.00997053 |
|  | 510 | N-Glycan biosynthesis | 2 | 0.01016496 |
|  | 53 | Ascorbate and aldarate  metabolism | 1 | 0.03425996 |
|  | 4012 | ErbB signaling pathway | 2 | 0.03842373 |
|  | 4912 | GnRH signaling pathway | 2 | 0.04097240 |

**Table S4 The statistically significant up-regulated and down-regulated pathways (p<0.05) and the number of associated genes in each pathway**.
