# Supplementary material for: Differential Gene Expression Profile in the Rat Caudal Vestibular Nucleus is Associated with Individual Differences in Motion Sickness Susceptibility
Source: PLoS One. 2015 Apr 24;10(4):e0124203. doi: 10.1371/journal.pone.0124203 (PMC4409317; doi:10.1371/journal.pone.0124203)
Supplement: S5 Table — (DOC) [file pone.0124203.s006.doc]

| Gene | Microarray  (MSS-Rot / nMSS-Rot) | Real-time PCR  (MSS-Rot / nMSS-Rot) |
| --- | --- | --- |
| nAchR α3 (Chrna3) | 1.25 | 1.61 |
| 5-HT_4_ R (Htr4) | 1.14 | 1.29 |
| NK_1_ R (Tacr1) | 1.15 | 1.36 |
| GABA_A_ α6 (Gabra6) | 0.56 | 0.53 |
| Olr81 (Olr81) | 2.11 | 2.07 |
| Shc1 (Shc1) | 0.71 | 0.74 |

**Table S5. Real-time PCR validation of microarray data for differentially expressed genes in the CVN.**
